# Dysregulation of Nutrient Sensing and CLEARance in Presenilin Deficiency

**DOI:** 10.1016/j.celrep.2016.02.006

**Published:** 2016-02-25

**Authors:** Kavya Reddy, Corey L. Cusack, Israel C. Nnah, Khoosheh Khayati, Chaitali Saqcena, Tuong B. Huynh, Scott A. Noggle, Andrea Ballabio, Radek Dobrowolski

**Affiliations:** 1 Federated Department of Biological Sciences, Rutgers University/New Jersey Institute of Technology, Newark, NJ 07102, USA; 2 Department of Molecular and Human Genetics, Baylor College of Medicine, Houston, TX 77030, USA; 3 Jan and Dan Duncan Neurological Research Institute, Texas Children Hospital, Houston, TX 77030, USA; 4 The New York Stem Cell Foundation Research Institute, New York, NY 10032, USA; 5 Telethon Institute of Genetics and Medicine (TIGEM), Pozzuoli, 80131 Naples, Italy; 6 Medical Genetics, Department of Translational Medicine, Federico II University, 80131 Naples, Italy

## Abstract

Attenuated auto-lysosomal system has been associated with Alzheimer disease (AD), yet all underlying molecular mechanisms leading to this impairment are unknown. We show that the amino acid sensing of mechanistic target of rapamycin complex 1 (mTORC1) is dysregulated in cells deficient in presenilin, a protein associated with AD. In these cells, mTORC1 is constitutively tethered to lysosomal membranes, unresponsive to starvation, and inhibitory to TFEB-mediated clearance due to a reduction in Sestrin2 expression. Normalization of Sestrin2 levels through overexpression or elevation of nuclear calcium rescued mTORC1 tethering and initiated clearance. While CLEAR network attenuation in vivo results in buildup of amyloid, phospho-Tau, and neurodegeneration, presenilin-knockout fibroblasts and iPSC-derived AD human neurons fail to effectively initiate autophagy. These results propose an altered mechanism for nutrient sensing in presenilin deficiency and underline an importance of clearance pathways in the onset of AD.

## Highlights

• Presenilin (PS)-knockout or AD mutations attenuate CLEAR network activity
• Amino-acid-sensing function of mTORC1 is dysregulated in PS-deficient cells
• Increase of cellular calcium or Sestrin2 re-regulates mTORC1 and CLEAR activity
• Dysregulated mTORC1 accounts for low autophagy in PS deficiency

## Graphical Abstract

**Figure:**
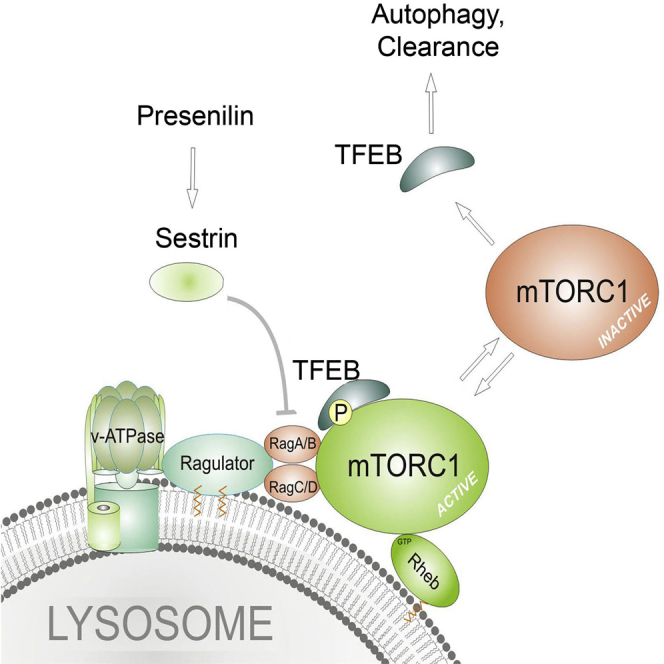

## Teaser

Reddy et al. find that the amino-acid-sensing function of mTORC1 is dysregulated in cells deficient in the AD-associated presenilin proteins. Constitutively active mTOR in these cells inhibits CLEAR network activity, leading to degeneration. Attenuation of the CLEAR network leads to the onset of AD-like pathophysiology in vivo.

## Introduction

Alzheimer disease (AD) is the most common neurodegenerative disorder of our time. Functional abnormalities of autophagosomes and lysosomes have been identified as some of the early pathological features in AD brains, preceding the hallmark deposits of amyloid and Tau tangles (Nixon and Yang, 2011). Enlargement of endosomal compartments containing amyloid precursor protein (APP) peptides (Takahashi et al., 2002), lysosomal deficits, and progressive accumulation of autophagic vacuoles are widely observed in AD human samples and corresponding mouse models (Cataldo et al., 1997, Nixon and Yang, 2011, Nixon et al., 2005). The link between AD and the lysosomal system is strengthened by observations that polymorphisms in several cathepsin genes increase the risk for AD (Bhojak et al., 2001, Papassotiropoulos et al., 1999) and deletions of lysosomal protease inhibitors cystatin B/C largely ameliorate symptoms in AD mouse models (Mi et al., 2007, Yang et al., 2011, Yang et al., 2014).

Impaired auto-lysosomal system, along with the consequential disruption of molecular trafficking and cellular signaling (Dobrowolski and De Robertis, 2011, Sorkin and von Zastrow, 2009, Taelman et al., 2010), is strongly linked to neurodegeneration (Komatsu et al., 2006, Lipinski et al., 2010, Nixon, 2013). Efficient (macro)autophagy is required to remove aggregated proteins and defective organelles, whose accumulation associates with a number of human diseases like AD, Parkinson disease, and amyotropic lateral sclerosis (Nixon, 2013). Autophagy is strictly dependent on lysosomal function that is driven by the nutritional status of the cell. Specifically, amino acids are sensed by lysosomes through a protein complex (vacuolar ATPase, Ragulator complex, and the Rag heterodimers A/B and C/D) that tethers the mechanistic target of Rapamycin complex 1 (mTORC1) to their membranes (Laplante and Sabatini, 2012, Nnah et al., 2015). The small GTPase Rheb (Ras homolog enriched in brain) activates mTORC1 on lysosomal membranes if TSC1/2 (tuberous sclerosis 1/2 complex) is inactivated by growth factor signaling (Inoki et al., 2003, Tee et al., 2003). Thus, mTORC1 activity is regulated by amino acid levels (as readily monitored by tethering of the complex to lysosomal membranes) and cellular signaling. Activity of mTORC1 has a direct effect on the biogenesis of lysosomes and autophagosomes through TFEB (transcription factor EB). TFEB is regulated by mTORC1 and positively regulates the activity of the CLEAR (coordinated lysosomal expression and regulation) gene network encoding for lysosomal and autophagosomal genes (Sardiello et al., 2009, Settembre et al., 2012). Under normal feeding conditions, active mTORC1 phosphorylates TFEB allowing it to remain in the cytoplasm. When cells starve, mTORC1 displaces from the lysosomal membranes, is no longer active, and is unable to phosphorylate TFEB that then translocates into the nucleus to directly bind to promoter elements containing the CLEAR sequence (Settembre et al., 2012, Settembre et al., 2013). This way, the mTORC1/TFEB pathway determines the activity of the auto-lysosomal system and the number of associated organelles. The mTOR kinase activity has been recently described as another risk factor for AD (Yates et al., 2013).

Altogether, these observations motivated us to study the regulation of the lysosomal mTORC1 pathway in early-onset familial AD (FAD) cells. FAD is caused by mutations in presenilin 1, 2 (PS1, 2) or APP. Besides the well-described functions of PS1, 2 in the γ-secretase complex, non-proteolytic functions of both proteins are currently discussed. In this line, PS1, 2 deficiency (absence of PS proteins or AD-associated mutation) is capable of impairing cellular calcium homeostasis of the endoplasmic reticulum (ER) and lysosomes (Coen et al., 2012, Popugaeva and Bezprozvanny, 2013, Tu et al., 2006) and its lysosomal function (Dobrowolski et al., 2012, Lee et al., 2010, Neely et al., 2011). Importantly, both alterations constitute pathogenic hallmarks of FAD with a possible inter-relationship (McBrayer and Nixon, 2013, Peric and Annaert, 2015). Although some of the mechanisms behind the auto-lysosomal dysfunction are known, such as PS-mediated pH alterations (Lee et al., 2010), it is very likely that additional factors contribute to autophagy malfunction in AD. Here, we studied lysosomal mTORC1 signaling in PS deficiency. We observed an attenuation of the CLEAR network activity and dysregulation of the regulatory transcription factor TFEB due to the inability of mTORC1 to properly sense amino acid availability in cells lacking PS proteins. The mTORC1 dysregulation was established by low nuclear/cytoplasmic calcium and consequently low Sestrin2 (Sesn2) levels, the increase of which normalized mTORC1 dynamics on lysosomal membranes and rescued CLEAR network activity. Attenuation of clearance in brain-specific TFEB knockout (KO) mice led to accumulation of total β-amyloid (Aβ), high Aβ42/40 ratio, paired helical filament (PHF) phospho-Tau (pTau), and neurodegeneration in hippocampal regions. In PS-deficient cells or induced pluripotent stem cell (iPSC)-derived human AD neurons, constitutively active mTORC1 failed to initiate autophagy.

Our results highlight the importance of PSs in the lysosomal pathway, and these data reveal PSs’ unanticipated role in mTORC1 amino acid-sensing and TFEB-mediated CLEAR network activity, the attenuation of which contributes to the onset of AD-like pathophysiology in vivo.

## Results

### The CLEAR Network Is Attenuated in PS Deficiency

**Figure 1:**
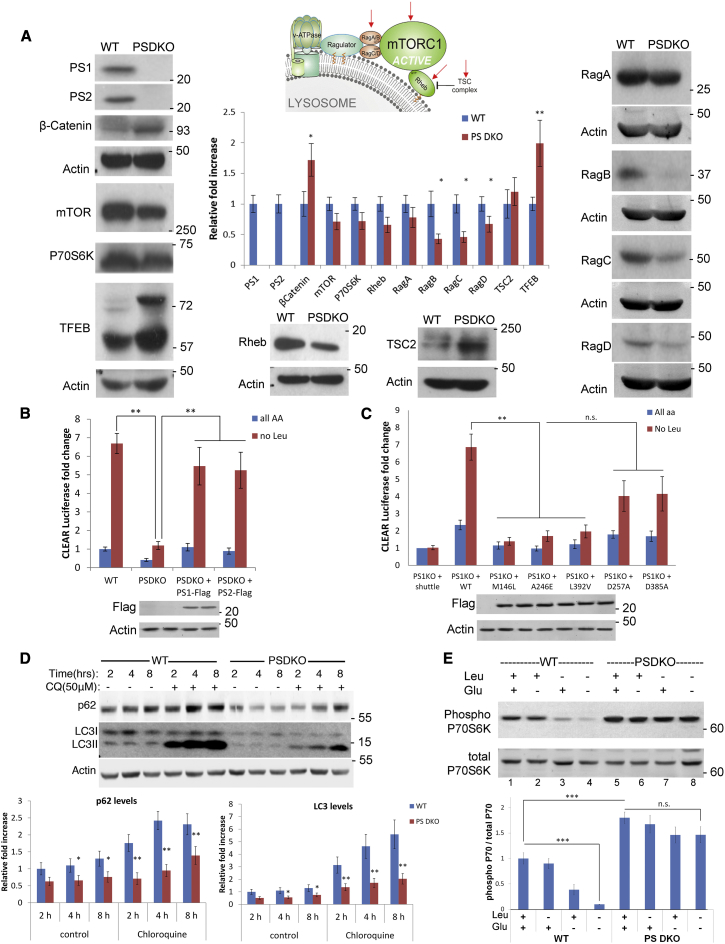
Mechanistic Target of Rapamycin Complex 1 (mTORC1) Is Deregulated in PS Deficiency, Attenuating the CLEAR Network Activity (A) Expression of the mTORC1 components involved in its tethering and activation at the lysosomes are lower (RagB and RagC, 0.4 ± 0.1; RagD, 0.6 ± 0.1; p = 0.05) in PSDKO cells, while levels of TFEB are higher. Bar graph represents quantitative analysis of protein levels assessed by immunoblot analyses. (B) PSDKO cells fail to efficiently activate the CLEAR gene network. Luciferase assay on WT and PSDKO cells transfected with the 4×CLEAR firefly luciferase reporter that were deprived of leucine overnight is shown. Data represent fold induction. Leucine-deprived PSDKO cells show a significant reduction of CLEAR reporter activity as compared to WT controls (WT 6.7 ± 0.1 versus PSDKO 1.2 ± 0.1; p = 0.01). This starvation response can be normalized by expressing exogenous PS1 or PS2-FLAG in PSDKO cells (5.4 ± 0.2 or 5.2 ± 0.2, respectively). Expression of FLAG-tagged PS1 and PS2 proteins is shown in representative immunoblot analyses; the order of samples is kept the same as in presented CLEAR luciferase assays. (C) FAD-associated PS1 point mutations were unable to rescue CLEAR attenuation. PS1-deficient cells were transfected with WT PS1 or FAD-linked PS1 plasmids. Unlike in the WT PS1 and γ-secretase loss-of-function mutants, expression of FAD-linked PS1 mutations failed to rescue CLEAR induction upon starvation (WT, 6.87 ± 0.76; M146L, 1.39 ± 0.22; p = 0.001; A246E, 1.69 ± 0.31; p = 0.005; L392V, 1.97 ± 0.37; p = 0.008; D257A, 4.03 ± 0.89; p = 0.08; D385A, 4.15 ± 0.99; p = 0.09; all p values state significance to WT controls as indicated with brackets). Expression of FLAG-tagged PS1 WT and mutant proteins is shown in representative immunoblot analyses; the order of samples is kept the same as in CLEAR luciferase assays. (D) Time course of lysosomal inhibition. WT and PSDKO cells were treated with Chloroquine (CQ) for the indicated time points and lysates assayed by immunoblotting for p62 and LC3I/II. In PSDKO cells, LC3 and p62 protein levels remained constantly lower than in WT controls. Note that PSDKO cells respond to CQ treatment and show a similar trend of p62 (at 2-hr time point, 1 ± 0.2 versus 0.63 ± 0.1; at 8-hr time point, 2.3 ± 0.3 versus 1.3 ± 0.2) and LC3 (at 2-hr time point, 1 ± 0.2 versus 0.51 ± 0.1; at 8-hr time point, 5.5 ± 1.1 versus 2.0 ± 0.4) accumulation as WT cells. Bar graph represents quantitative analysis of protein levels assessed by immunoblot analyses. (E) mTORC1 activity represented in immunoblots for phsopho-P70S6K. While mTORC1 activity is attenuated in control cells under baseline (lane 1, WT, 1 ± 0.1 versus lane 5, PSDKO, 1.8 ± 0.11) and amino acid starvation conditions (lane 4, Glu-, Leu-deficient medium containing 10% dFBS, respectively), mTORC1 is insensitive to leucine or glutamine withdrawal and constitutively active in PSDKO cells (lane 8, WT, 0.1 ± 0.002 versus PSDKO 1.47 ± 0.15; p = 0.001). Data are represented as mean ± SEM. Bar graph represents quantitative analysis of protein levels assessed by immunoblot analyses. Data are represented as mean ± SEM. See also Figure S1.

Defective autophagy induction and flux have been attributed to the absence of PS1 protein. Conflicting reports have implicated lysosomal pH in the autophagic defects of PS1-deficient cells due to the faulty maturation of v-ATPase and homeostatic calcium changes (Coen et al., 2012, Lee et al., 2010, Zhang et al., 2012). In an effort to better understand the lysosomal mTORC1 function in PS-deficient cells, we began our studies using the readily available PS 1, 2 double KO (PSDKO) mouse embryonic fibroblasts (MEFs) (Wakabayashi et al., 2009), then expanded our analyses to human FAD fibroblasts and iPSC-derived human neurons. As mTORC1 has yet to be characterized in PS deficiency, we decided to determine the expression levels of proteins associated with the complex. All proteins belonging to the Rag complex were lower in expression, with RagB being the most significantly reduced (Figure 1A). Expression of mTOR and overall levels of the mTORC1-associated activator Rheb proteins also were significantly reduced in PSDKO cells. In contrast, we found elevated levels of the mTORC1 inhibitor TSC2 and β-catenin in PSDKO cells, confirming results published by us and others (Dobrowolski et al., 2012, Kang et al., 2002). Increased expression of TFEB and its phosphorylated form was constantly detected in PSDKO cells (Figure 1A). Importantly, a re-expression of PS1 and PS2 in PSDKO cells normalized the levels of Rag B-D, β-catenin, and TFEB (Figure S1D).

Next we determined whether these mTORC1-related differences in PS-deficient cells would have an impact on CLEAR gene network activity and autophagy induction. For this purpose, we transiently transfected PSDKO cells with previously published 4×CLEAR-firefly luciferase reporter (Sardiello et al., 2009) and appropriate constitutive Renilla-luciferase constructs. In amino acid starvation experiments, we found strongly reduced activation of the CLEAR reporter expression upon leucine deprivation in PS-deficient cells, which was normalized by overexpressing either PS1 or PS2 construct (Figure 1B), further underlining the redundancy of the two PS isoforms and the importance of γ-secretase-independent function of these proteins. Since PS loss does not entirely represent disease conditions, we performed a PS1KO rescue experiment in which PS1 wild-type (WT) or AD-associated PS point mutations were overexpressed; the PS1 M146L, A246E, and L392V mutations were unable to rescue the observed CLEAR network attenuation. Notably, the secretase loss-of-function mutations D257A and D385A showed a tendency to rescue PS loss in these experiments (Figure 1C). Treatment of cells with γ-secretase inhibitors did not show significant effects on CLEAR network activity (Figure S1C), indicating that the observed CLEAR-associated phenotype is possibly secretase independent.

Western blot and qPCR analyses of autophagy-associated genes further confirmed the attenuation of the CLEAR network. We found the low levels of LC3AB and p62 and the ability of PSDKO cells to induce autophagy to be significantly lower in our flux assays (Figures 1D and S1A). To further confirm these analyses, we used iPSC-derived human neurons depleted of PS1 (Figure S1B). Importantly, overexpression of exogenous PS1-FLAG normalized the autophagy flux levels in these isogenic cultures; in all other experiments, cells with low passage numbers were used. Furthermore, in microscopic autophagy flux assays using eGFP-mRFP-LC3 (Kimura et al., 2007), we found an attenuation of baseline autophagy as indicated by a moderate buildup of yellow LC3 puncta in PSDKO cells (Figure S1F).

To ascertain the activity of mTORC1 under amino acid starvation conditions, we cultured PSDKO cells in leucine- deficient RPMI media containing dialyzed fetal bovine serum (FBS), as recently described for other KO MEF cell lines (Peng et al., 2014). In PSDKO cells, mTORC1 activity was largely insensitive to amino acid starvation, as determined in pP70 immunoblot analyses (Figure 1E), whereas mTOR protein stability was increased in pulse-chase assays (Figure S1E). These findings motivated us to investigate the dynamics of mTOR upon amino acid withdrawal in PS-deficient cells.

### Amino Acid Sensing of mTORC1 Is Impaired While TSC2 Dynamics Remain Unchanged in PS Deficiency

**Figure 2:**
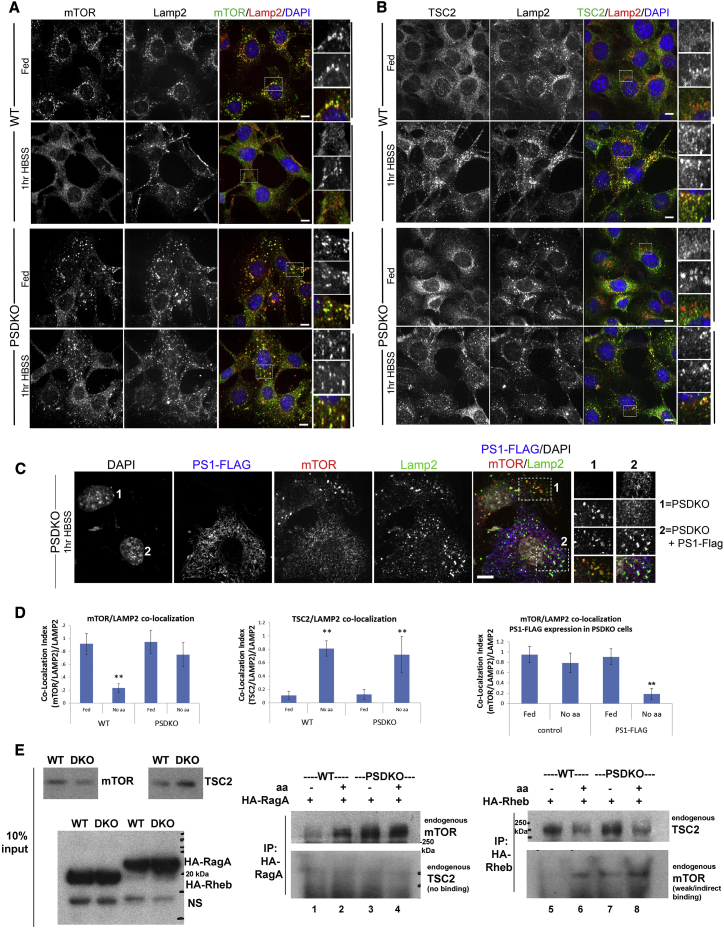
Defective Amino Acid Sensing of mTORC1 in PSDKO Cells Is Mediated by Excessive RagA Binding and Independent of TSC2 Localization (A) mTOR remains localized to lysosomes upon nutrient deprivation in PSDKO cells. WT and PSDKO cells were starved in amino acid-deficient medium (HBSS) for 1 hr. The localization of mTOR and LAMP2 was determined by immunostaining. Insets depict selected fields that were magnified. (B) TSC2 localizes normally to lysosomes upon nutrient deprivation in PSDKO cells. WT and PSDKO cells were starved, as above. The localization of TSC2 and LAMP2 was determined by immunostaining. Insets depict selected fields that were magnified. (C) The excessive lysosomal mTOR tethering under amino acid starvation conditions in PSDKO cells (cell 1) can be reversed by expression of exogenous PS1-FLAG (cell 2). (D) Quantification of lysosomal mTOR or TSC2 localization presented in (A)–(C) is shown. mTOR/LAMP2 co-localization in WT, 0.23 ± 0.06 versus PSDKO, 0.75 ± 0.2; p = 0.01; TSC2/LAMP2 co-localization in WT, 0.8 ± 0.05 versus PSDKO, 0.7 ± 0.05; p = 0.1; mTOR/LAMP2 co-localization in PSDKO (control), 0.78 ± 0.2 versus PSDKO + PS1-FLAG, 0.18 ± 0.1; p = 0.01. (E) Co-immunoprecipation analyses of endogenous mTOR and TSC2 proteins to HA-RagA and HA-Rheb show a sustained binding of mTOR proteins to RagA and Rheb in PSDKO cells, whereas TSC2 protein-binding dynamics were similar in both cell lines under starvation versus fed conditions. All scale bars, 10 μm. Data are represented as mean ± SEM. See also Figure S2.

Amino acid starvation diffuses the otherwise tethered mTORC1 from the lysosomal membranes to the cytoplasm, whereas the inhibitory TSC2 re-localizes from the cytoplasm to the lysosomal membranes to inhibit Rheb (Demetriades et al., 2014, Menon et al., 2014, Sancak et al., 2010). Since the levels of the RagB to RagD proteins are lower in PSDKO cells (Figure 1A), we examined whether mTOR dynamics are changed upon nutrient deprivation in PSDKO cells. In WT cells, mTOR co-localized to LAMP2-positive vesicles in nutrient-rich conditions (Figure 2A). Upon 1 hr of amino acid withdrawal (Hank’s balanced salt solution [HBSS]), mTOR diffused into the cytoplasm. Once the autophagy pathway is induced and amino acid levels increase as a result of protein breakdown, mTOR tethers back to lysosomal membranes in a RagA-dependent manner. In PSDKO cells, LAMP2-positive vesicles were more dispersed throughout the cytoplasm and larger than in WT cells. Under nutrient-/amino acid-rich conditions, mTOR localizes to lysosomes (Figure 2A and quantifications in Figure 2D), and the vast majority of mTOR remained localized to lysosomes in amino acid starvation. To further test the PS dependence of lysosomal mTOR tethering, we overexpressed PS1-FLAG in PSDKO cells (Figure 2C). Under amino acid starvation conditions, mTOR dispersed from lysosomes only in cells expressing exogenous PS1-FLAG (cell 2 in Figure 2C and quantifications in Figure 2D). In co-immunoprecipitation analyses, we observed an excessive binding of endogenous mTOR protein to HA-RagA under amino acid starvation conditions specifically in PSDKO cells (Figure 2E, lanes 3 and 4), further indicating a defect in the RagA-mediated amino acid sensing of these cells.

Since TSC2 has been described to be required for complete release of mTORC1 from the lysosomes upon amino acid starvation (Demetriades et al., 2014), we next investigated TSC2 dynamics in WT and PSDKO cells in the same starvation assays. We hypothesized that TSC2 fails to be recruited to the lysosomes under nutrient deprivation in PSDKO cells and thus is unable to fully release mTOR from lysosomes. Similar experiments as those shown in Figure 2A were conducted and assessed for TSC2 localization with LAMP2. However, no differences in TSC2 localization could be detected between PSDKO and WT cells (Figure 2B and quantifications in Figure 2D). Amino acid deprivation for 1 hr re-localized TSC2 to LAMP2 vesicles in both WT and PSDKO cells. Longer nutrient deprivation (4 hr) kept TSC2 on the LAMP2 vesicles in both cell types (Figure S2), even though mTOR re-localized to LAMP2 vesicles under prolonged starvation. We also show that refeeding both WT and PSDKO cells with complete media after a brief amino acid starvation was sufficient to disperse TSC2 back to the cytoplasm (Figure S2B). Furthermore, dissociation of TSC2 from HA-Rheb in fed conditions was comparable between both, WT and PSDKO cells (Figure 2E, lanes 6 and 8). We conclude that the response of TSC2 to starvation and its shuttling are unchanged, while mTORC1 dynamics, and hence amino acid sensing through the mTOR tethering RagA proteins, are highly deregulated in PS deficiency.

### Increasing Cellular Calcium Normalizes Sesn2 Levels and Rescues CLEAR Activity and mTORC1 Sensing in PS-Deficient Cells

PS proteins have been described to exist as weak calcium leak channels or as regulatory proteins of ER-resident calcium channels (Tu et al., 2006). PS-deficient cells hold less calcium in the cytoplasm (Tu et al., 2006). Therefore, we asked if calcium plays a role in regulating the defective mTORC1 diffusion phenotype in PSDKO cells. We used Calcium Ionophore (CaI, A23187) to increase overall calcium levels in the cytoplasm and other cellular compartments by loading calcium from the extracellular environment (Resendez et al., 1985). WT and PSDKO cells were pretreated with CaI for 4 hr and then amino acid deprived (1 hr HBSS) to assay for mTOR dynamics under starvation. Increasing cytoplasmic calcium dispersed mTOR from LAMP2-positive vesicles under fed and amino acid starvation in both cell lines, thus, rescuing the mTOR-tethering phenotype in PSDKO cells (Figures S3A and S3B).

**Figure 3:**
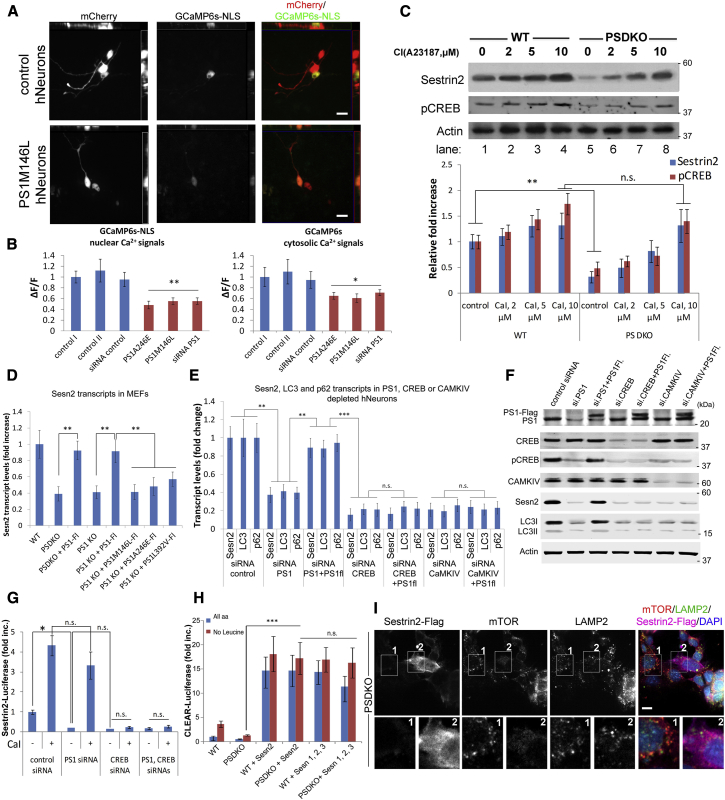
Low Nuclear Calcium Levels Are Responsible for Low Sesn2 Levels and mTOR Dysregulation in PS-Deficient Cells (A and B) iPSC-derived human neurons expressing GCaMP6s-NLS or GCaMP6s used to determine nuclear and cytosolic calcium levels. mCherry was used to visualize neuronal projections, which were largely excluded when GCaMP6s-NLS vectors were used. Images show orthogonal maximal intensity projections of x/y, x/z, and y/z perspectives. PS1M146L, PS1A246E, and PS1 siRNA-depleted mutant neurons show a significant reduction of cytosolic and more significant reduction of nuclear calcium levels (for cytosolic calcium levels, PS1M146L 0.61 ± 0.07, PS1A246E 0.65 ± 0.06, siRNA PS1 0.71 ± 0.05; p = 0.05; for nuclear calcium levels, PS1M146L 0.55 ± 0.06, PS1A246E 0.48 ± 0.07, siRNA PS1 0.52 ± 0.06; p = 0.01). (C) Elevated cellular calcium increases the otherwise low Sesn2 levels in PSDKO cells. WT and PSDKO cells were treated with increasing concentrations of Calcium Ionophore (CaI, 2–10 μM) overnight followed by analyses for pCREB and Sesn2 protein levels (baseline pCREB levels, WT 1 ± 0.14 versus PSDKO 0.47 ± 0.11; p = 0.01; pCREB after CaI, 10 μM, WT 1.7 ± 0.2 versus PSDKO 0.89 ± 0.3; p = 0.1; baseline Sesn2 levels, WT 1 ± 0.1 versus PSDKO 0.3 ± 0.1; p = 0.01; Sesn2 after CaI, 10 μM, WT 1.3 ± 0.3 versus PSDKO 1.3 ± 0.2; p = 0.01). (D) PS regulates Sesn2 transcription. Sesn2 transcript levels are low in PS KO cells and increase with expression of exogenous PS1WT-FLAG constructs, while AD-associated PS mutations do not (PSDKO + PS1-FLAG, 0.92 ± 0.1 and PS1KO + PS1-FLAG, 0.91 ± 0.1 versus PS1M146L, 0.41 ± 0.1, A246E, 0.48 ± 0.2, L392V, 0.6 ± 0.1; p = 0.01). (E and F) Sesn2 expression is dependent on PS1, CREB, and CaMKIV. qPCR analyses in (E) show low Sesn2, LC3, and p62 mRNA levels when PS1, CREB, or CaMKIV are depleted in human neuronal cultures (for Sesn2 levels, siRNA control 1 ± 0.1 versus siRNA PS1 0.37 ± 0.1, siRNA CREB 0.2 ± 0.1, and siRNA CaMKIV 0.2 ± 0.1; p = 0.001). Expression of exogenous PS1WT-FLAG rescues LC3, p62, and Sesn2 levels only in PS1-depleted neurons (0.89 ± 0.1; p = 0.1 to control). Immunoblot analyses in (F) show the expression of the samples on protein level; all used siRNA pool-depleted protein levels to at least 40% of their control levels. (G) Sesn2 promoter activity measured in Sesn2-luciferase reporter assays is significantly attenuated in PS1-depleted human neurons (control, 1 ± 0.1 versus PS1siRNA, 0.2 ± 0.01; p = 0.04; left bracket) and can be normalized to control levels (control, 4.32 ± 0.5 versus PS1siRNA, 3.3 ± 0.7; p = 0.1; left bracket) in a CREB-dependent manner. (H) Overexpression of Sesn1,2,3 or Sesn2 alone rescues the attenuated CLEAR induction in PSDKO cells in CLEAR-luciferase reporter assays (1.35 ± 0.15 versus 17.3 ± 3.3; p = 0.001; left bracket). (I) Overexpression of Sesn2-FLAG in PSDKO cells disperses the otherwise tethered mTORC1 to lysosomes. PSDKO cell is labeled with 1 and Sesn2-FLAG-expressing cell is depicted as 2. mTOR diffusion is visible in single-channel images of cell 2 (PSDKO, 0.88 ± 0.3 versus PSDKO + Sesn2-FLAG, 0.26 ± 0.1; n = 200; p = 0.05). Data are represented as mean ± SEM. All scale bars, 10 μm. Data are represented as mean ± SEM. See also Figure S3.

Total cytosolic and nuclear calcium concentrations were ascertained in three control and three PS1-deficient (M146L-, A246E-, and small interfering RNA (siRNA)-depleted) human neuron lines using the genetically encoded GCaMP6s (Chen et al., 2013) and GCaMP6s-NLS sensors (Hagenston and Bading, 2011). We found low cytosolic calcium levels in PS-deficient human neurons (Figures 3A and 3B), and we confirmed the findings of other groups in other cell lines (Tu et al., 2006). Furthermore, we found that nuclear GCaMP6s-NLS fluorescence, hence nuclear calcium levels, was reduced in PS-deficient neurons (Figures 3A, 3B, and S3D). To assess the effects of nuclear calcium signaling in cells, we performed immunoblot analyses of CaI-treated cells for 4 hr. CaI significantly elevated the levels of pCREB (cAMP response element-binding protein) and Sesn2, the recently described mTORC1-associated protein (Chantranupong et al., 2014, Peng et al., 2014), strongly in PSDKO cells and to a lower extent in WT cells (Figure 3C). We confirmed the PS-dependent Sesn2 expression in qPCR analyses (Figures 3D and S3E) using PS1 and PS1,2 DKO MEFs. Levels of Sesn2 mRNA significantly decreased in both PS KO cell lines were normalized with overexpression of PS1WT-FLAG, but they did not change when FAD-associated PS1 mutations were expressed. In iPSC-derived human neurons depleted of PS1, Sestrin levels were lower similar to MEFs and could be rescued with hPS1-FLAG overexpression (Figures 3E and 3F). Given our results indicating the involvement of nuclear calcium signaling in PS deficiency, we tested whether Sesn2 is a CaMKIV and CREB target gene. Indeed, Sesn2 expression was dependent on CaMKIV and CREB expression and could not be rescued by an overexpression of PS1-FLAG alone, indicating that PS function on Sesn2 is upstream of the CaMKIV/pCREB signaling (Figures 3E and 3F). In all experiments, Sesn2 mRNA and protein levels strongly correlated with the expression levels of the autophagosomal marker LC3 and autophagosome cargo marker p62. Furthermore, Sesn2 promoter activity was significantly lower in PS1-depleted neurons and could be increased by Calcium Ionophore in a CREB-dependent manner (Figure 3G).

To further confirm the role of Sesn2 in neuronal mTOR signaling, we analyzed the binding of endogenous mTOR proteins to HA-RagA in iPSC-derived human neurons (Figure S3F). Under amino acid starvation conditions, binding of mTOR to HA-RagA was strongly increased when PS1 or Sesn2 was depleted (Figure S3F, lane 2 versus lanes 4 and 6), while overexpression of Sesn2-FLAG decreased the mTOR-RagA binding in both conditions and cell lines (lanes 7–10). These data indicate the potential involvement of nuclear calcium signaling on Sestrin expression and neuronal homeostasis (Bading, 2013).We further asked whether increasing Sestrin levels would impact the CLEAR gene network activity. Exogenous Sesn2 or Sesn1, 2, and 3 were capable of increasing CLEAR luciferase reporter levels (Figure 3H) and dispersing the otherwise tethered mTOR from lysosomes in PSDKO cells (Figure 3I). Importantly, both treatments, CaI and Sestrin overexpression, largely eliminated the differences in CLEAR network activity between WT and PSDKO cells.

### TFEB-Driven Clearance Functions Are Impaired in PS-Deficient Cells

**Figure 4:**
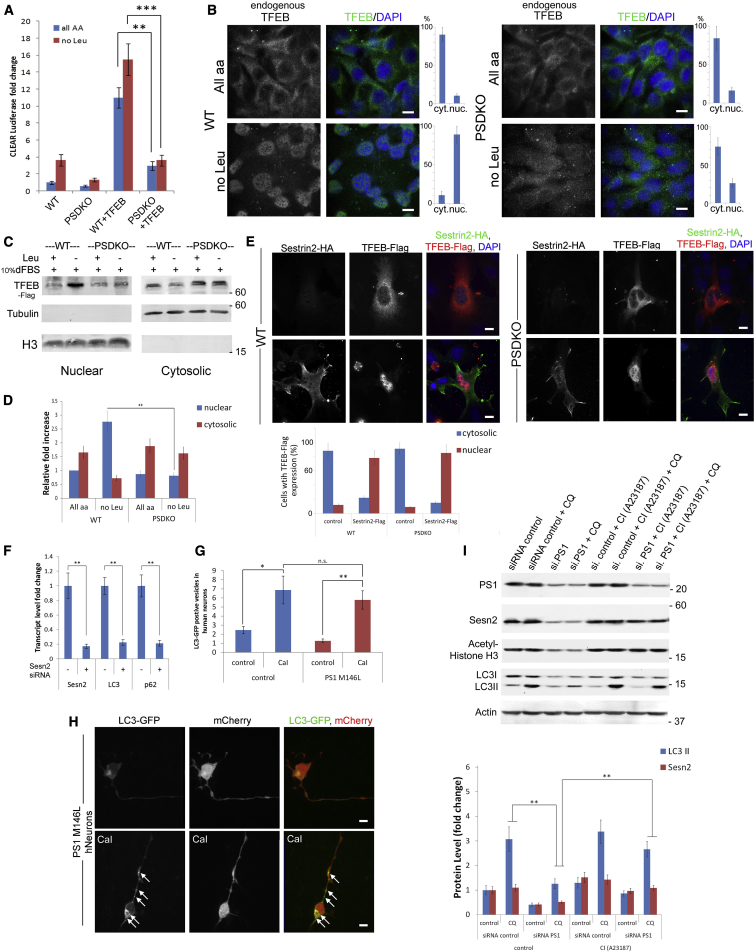
TFEB-Driven Clearance Functions Are Impaired Due to Low Sesn2 Levels in PS-Deficient Cells (A) Unlike in WT cells, moderate expression of exogenous TFEB-FLAG proteins in PSDKO cells fail to sufficiently induce CLEAR activation. (10.9 ± 1.7 versus 3.0 ± 0.5; p = 0.03; left bracket, all aa; 15.5 ± 1.8 versus 3.6 ± 0.6; p = 0.008; right bracket, no leucine). (B) TFEB dynamics are impaired in PSDKO cells. Whereas TFEB localizes to the cytosol during feeding and enters the nucleus upon starvation in WT cells (nuclear TFEB, 10% ± 2.7% versus 89% ± 10.8%; p = 0.004), localization of TFEB does not significantly change before or after leucine withdrawal in PSDKO cells (nuclear TFEB, 16% ± 4.6% versus 26% ± 5.9%; p = 0.1). (C and D) Cytoplasmic nuclear fractionation analyses show impaired TFEB nuclear re-localization in PSDKO cells (PSDKO all aa versus no Leu, 0.87-fold ± 0.12 versus 0.81-fold ± 0.11; p = 0.1). (E) Sesn2-HA overexpression rescues the TFEB-FLAG nuclear re-localization in PSDKO cells (nuclear TFEB in Sesn2-FLAG-positive cells, 78% ± 10.9% in WT versus 85% ± 11.9% in PSDKO cells). (F) Transcriptional efficiency of LC3 and p62 dependents on Sesn2 expression (Sesn2, 0.17 ± 0.03; LC3, 0.22 ± 0.04; p62, 0.21 ± 0.03; p = 0.01, respectively). (G) Quantification of LC3-GFP-positive vesicles in human neurons after CaI treatment (Control, 6.8 ± 1.5, 14.1 ± 2.8; p = 0.05, 0.01, respectively; PS1M146L, 4.4 ± 2.1, 12.5 ± 2.6, p = 0.06, respectively). (H) The number of LC3-GFP vesicles increases in PS1M146L human neurons after CaI treatment (see white arrows). (I) Immunoblot analyses including autophagy flux assays in PS1-depleted, CaI-treated human neurons show a reduction of LC3II and Sesn2 levels in PS1-depleted cells after CQ treatment (LC3, control, 3.1 ± 0.2 versus LC3, si.PS1, 1.26 ± 0.2; p = 0.01; Sesn2, control, 1.1 ± 0.1 versus Sesn2, si.PS1, 0.4 ± 0.1; p = 0.01), while a treatment with CI equilibrated the LC3 and Sesn2 levels of both cell lines to each other (CI, LC3, control, 3.4 ± 0.48 versus LC3, si.PS1, 2.7 ± 0.3; p = 0.1; Sesn2, control, 1.4 ± 0.2 versus Sesn2, si.PS1, 1.1 ± 0.1; p = 0.1). All scale bars, 10 μm. Data are represented as mean ± SEM. See also Figure S4.

TFEB regulates the CLEAR gene network and its activity was described to be associated with health and human disease (Füllgrabe et al., 2014, Settembre et al., 2013). Since TFEB is regulated by mTORC1, which we found to be deregulated in PS deficiency, we asked whether attenuation of the CLEAR gene network may be attributed to a deregulation of this transcription factor. The first indication of a deregulated mTOR/TFEB pathway was provided by the CLEAR reporter assays. We observed that moderate expression of exogenous TFEB-FLAG proteins in PSDKO cells failed to significantly induce CLEAR activation, while expression of the same amount of TFEB-FLAG strongly induced activity of the network in WT cells (Figure 4A). We therefore analyzed the localization of endogenous TFEB proteins in PS-deficient and control cells under fed and leucine-deprived conditions. We observed that TFEB was not properly re-localized into the nucleus in leucine-starved PSDKO cells, whereas nuclear TFEB was readily observed in starved WT cells (Figures 4B and 4C). No significant changes to TFEB localization could be found in PSDKO cells starved with leucine-deficient RPMI media (containing 10% dFBS).

These data further indicate a deregulation of mTORC1 activity and phosphorylation of its protein substrates, like TFEB, in PS-deficient cells. Hence, we next overexpressed Sesn2-FLAG to analyze the effect of mTORC1 activity on TFEB dynamics. We predicted that exogenous Sestrin expression would inhibit mTORC1-mediated phosphorylation through mTOR detachment from lysosomal membranes. In both WT and PSDKO lines, overexpression of Sesn2-FLAG was sufficient to re-localize endogenous TFEB into the nucleus (Figure 4E), probably due to its inhibitory effect on mTOR-RagA binding (Figure S3F) and, subsequently, its activity (assessed later on in our study).

Since elevation of cellular calcium or Sestrin level restored amino acid sensing in PSDKO cells, we analyzed whether autophagy-related TFEB target genes, LC3 and p62, are increased with these treatments. We found that CaI increased transcript levels of Sestrin, LC3, and p62. We furthermore found that Sesn2 expression for itself was required for sufficient LC3 and p62 transcription (Figure 4F). Treatment of PS-deficient (M146L or siRNA PS1-depleted) human neurons with CaI increased the number of LC3-GFP-positive vesicles (Figures 4G and 4H) and functional autophagy, as assessed in flux assays (Figure 4I). Interestingly, PS1-depleted neurons showed lower Histone H3 acetylation and Sesn2 and LC3 expression, while CaI was capable of increasing the levels of these proteins and inducing autophagy, as seen in increased LC3II levels after Chloroquine (CQ) treatment. Altogether these data underline the importance of the nuclear calcium and Sesn2 functions in autophagy.

### Attenuation of Cellular Clearance Leads to AD-like Phenotypes and Degeneration

**Figure 5:**
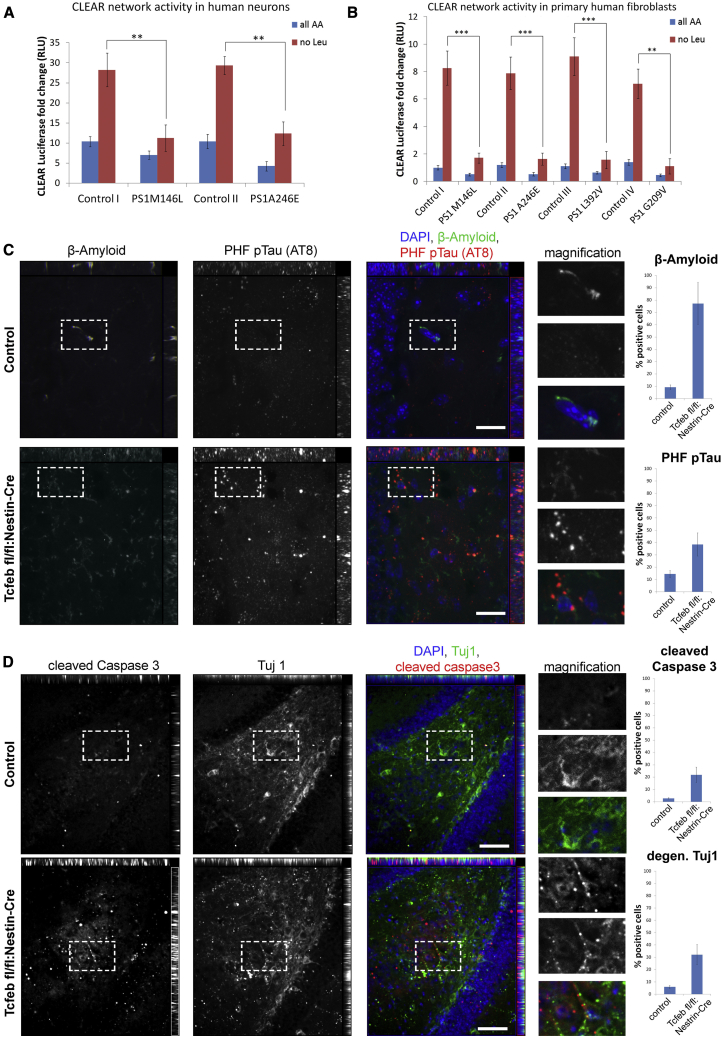
Cellular Clearance Is Attenuated in AD Cells and Leads to AD-like Phenotypes In Vivo (A) iPSC-derived human neurons were used for CLEAR network reporter assays. Neurons carrying the FAD mutations (PS1M146L and PS1A246E) failed to efficiently express the CLEAR luciferase reporter under leucine starvation conditions (M146L, 11.2 ± 3.3, A246E, 12.3 ± 2.9 versus control I, 28.3 ± 6.7, control II, 29.4 ± 2.2; p = 0.01). (B) Defective activation of the CLEAR network activity upon leucine withdrawal in FAD hFibroblasts carrying four different PS1 mutations and compared to four control cell lines (M146L, A246E, L392V, and G209V, 1.7 ± 0.3, 1.6 ± 0.4, 1.5 ± 0.6, and 1.1 ± 0.5, respectively, versus control lines, 8.3 ± 1.2, 7.9 ± 1.2, 9.1 ± 1.4, and 7.1 ± 1.0; p = 0.001). (C) Attenuation of the CLEAR network in vivo: Tcfeb fl/fl:Nestin-Cre mouse brains show an accumulation of Aβ and PHF pTau (AT8) in the hippocampal regions (77.2% ± 16.9%, p = 0.001 or 38.4% ± 9.3%, p = 0.003, respectively). Images show orthogonal maximal intensity projections of x/y, x/z, and y/z perspectives. (D) An accumulation of the apoptotic marker cleaved caspase 3 and an irregular spread of the neuronal tubulin (Tuj1) in same hippocampal regions of the Tcfeb fl/fl:Nestin-Cre mutant mice were detected (21.8% ± 6.1%, p = 0.005 and 32.1% ± 8.1%, p = 0.004, respectively). Images show orthogonal maximal intensity projections of x/y, x/z, and y/z perspectives. All scale bars, 20 μm. Data are represented as mean ± SEM. See also Figure S5.

To test whether the mechanism presented here is relevant to human AD, we next determined the activity of the CLEAR network in human cells (fibroblasts and neurons) and the consequences of CLEAR network attenuation in vivo. Human fibroblasts (M146L, A246E, L392V, and G209V; Coriell Institute) as well as iPSC-derived human AD neurons (PS1M146L and PS1A246E) showed small but significant attenuation of CLEAR luciferase reporter activity under baseline (fed conditions), whereas starvation conditions showed an obvious defect in the initiation of the CLEAR network (Figures 5A and 5B). We found the expected mTORC1 dynamics in control human fibroblasts: mTOR proteins appeared diffusely localized in the cytoplasm and only a small amount of mTOR seemed still bounded to the lysosomal membrane under starvation conditions. In FAD cells, however, most of the cellular mTOR was localized to the lysosomal compartment even under starvation conditions (Figure S5D).

To assess the consequences of an attenuation of the CLEAR gene network, we analyzed the conditional deletion of TFEB in the mouse brain (Tcfeb flox/flox:Nestin-Cre). Transcripts isolated from the whole-brain lysates showed a strong reduction of Tcfeb mRNA levels as well as an attenuation of TFEB target gene expression: p62 and LC3 (Figure S5C). Specifically, the hippocampal region of 2-month-old mice showed a significant increase of total Aβ and PHF pTau buildup in the absence of TFEB (Figure 5C). Biochemical analyses of total Aβ and Aβ42/40 indicated a trend for total Aβ and a significant increase of the Aβ42/40 ratio in TFEB-deficient whole-brain lysates (Figures S5A and S5B). While minimal levels of the apoptotic marker cleaved caspase 3 were found in control animals, TFEB-deficient brains showed a significant increase of apoptotic cells with punctuated neuronal tubulin (Tuj1), indicative of axonal degeneration (Figure 5D).

These results imply an important role of the mTOR/TFEB-driven clearance pathways in neuronal homeostasis, and they associate these pathways directly with the onset of neurodegenerative pathophysiology like that seen in AD.

## Discussion

AD is characterized by a complex pathophysiology involving the buildup of two main neurotoxic protein aggregates composed of Aβ or hyperphosphorylated Tau. Abnormalities in the auto-lysosomal system are discussed as the early features preceding the well-known hallmarks (Nixon and Yang, 2011). Defective auto-lysosomal system can directly impact molecular trafficking, cellular signaling, and clearance, leading to disease onset in the aging brain. Early-onset FAD is caused by mutations in APP or PS. PS deficiency has been associated with lysosomal function through the maturation of vATPase complex (Lee et al., 2010) or independent of such (Coen et al., 2012, Neely et al., 2011, Zhang et al., 2012).

In the research presented here, we showed that the levels, activity, and dynamics of the major cellular kinase mTORC1—as well as its functions in autophagosomal pathways—are dysregulated in PS deficiency due to its excessive tethering to lysosomal membranes. We found the key transcription factor for lysosomal biogenesis, TFEB, to be highly phosphorylated in PSDKO cells (Figure 1A) and human AD neurons. High levels of p-TFEB correlated with attenuated CLEAR gene network activity (Figures 1B, 1C, 5A, and 5B) and reduced expression levels and, consequently, function of the autophagosomal markers LC3 and p62 (Figures 1D, S1B, 3F, 4I, 6D, and 6E). Using exogenous, fluorescent LC3 biosensors, autophagy flux seemed moderately attenuated (Figure S1F); however, we would like to note that LC3 is a target gene of TFEB whose activity is attenuated in PS-deficient cells. Overexpression of exogenous LC3 in these cells likely changes the autophagic phenotype of PS-deficient cells. We hypothesized that the attenuated CLEAR gene network activity explains the lysosomal and autophagosomal inhibition found in PS deficiency and early-onset FAD. All tested PS mutations associated with AD (but not γ-secretase) showed a significant reduction of CLEAR gene network activity to starvation cues (Figures 1B, 1C, 5A, and 5B). These data seemingly disagree with a study published by Zhang et al. (2012) stating increased lysosomal biogenesis in PSDKO brains, itself objecting to studies showing that increased TFEB-mediated clearance decreases pTau and amyloid levels in AD mouse models (Polito et al., 2014, Xiao et al., 2014), not contributing to their buildup. The reason for these discrepancies are unknown; however, they could be due to the use of whole-cell lysates and contamination through other, non-neuronal cells.

Lysosomal localization of mTORC1 is crucial for its activity and is determined by the availability of amino acids (Sancak et al., 2010). Localization of the Rheb inhibitors TSC1/2 determines the activity of the complex and, thus, the phosphorylation state of its protein substrates. We therefore closely monitored the dynamics of mTOR and TSC2 as well as mTORC1 activity in PS-deficient cells. Depriving control cells of amino acids largely inhibited mTORC1 activity in controls, while PS-deficient cells, MEFs, and human neurons maintained an active kinase complex through a strong binding of mTOR to RagA (Figures 1E, 2E, and S3F). The strong binding of mTOR to RagA correlates with its constitutive tethering to lysosomal membranes (Figure 2A), whereas TSC2 re-localized from the cytoplasm to the lysosomal compartment in fed or amino acid-starved conditions, respectively. These readily reproducible findings indicate that the amino acid-sensing pathways in PS-deficient cells are largely dysregulated and that TSC2 can tether to the lysosomal membranes without displacing mTORC1. Since both proteins, mTOR and TSC2, localize to the lysosomal compartment under starvation conditions in PS-deficient cells (Figure S2A), our data support the model proposed by Menon et al. (2014) in which TSC2 tethers to lysosomes via Rheb while mTORC1 remains bound to the Rags on the lysosomal surface (Figures 2A and 2B; Sancak et al., 2008, Sancak et al., 2010).

**Figure 6:**
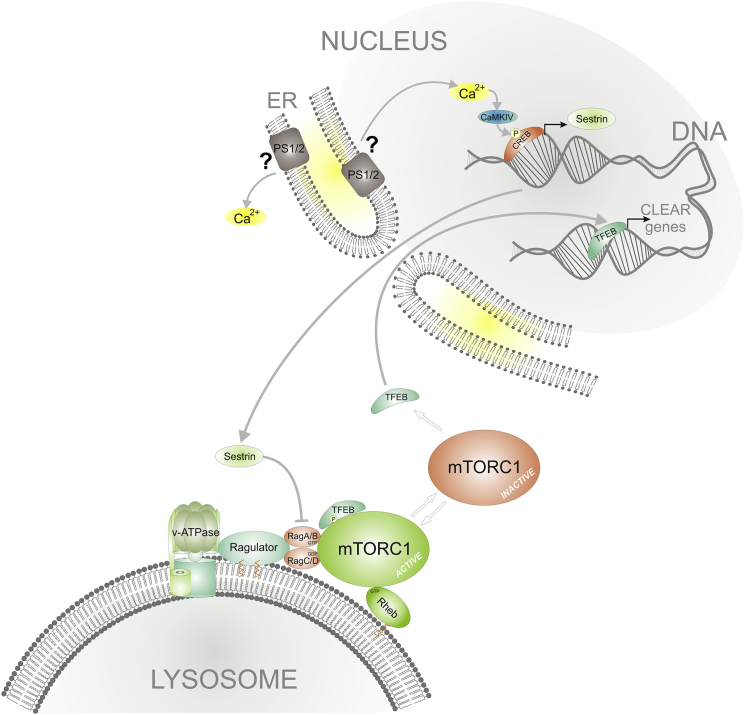
Model of PS Function in mTORC1/TFEB Signaling through Regulation of Calcium/Sestrin Levels PSs as or part of a calcium channel on ER membranes impact nuclear calcium levels that regulate Sesn2 gene expression. Sufficient levels of Sesn2 proteins promote mTORC1 release from lysosomal membranes, this way inhibiting its activity. Non-phosphorylated TFEB re-locates into the nucleus to increase CLEAR network activity and cellular clearance, antagonizing buildup of toxic protein aggregates.

We found that low Sesn2 levels are responsible for the excessive mTORC1 tethering in PS-deficient cells (MEFs and iPSC-derived human neurons) and that the reduction of Sesn2 expression is a consequence of the low nuclear calcium and CaMKIV/pCREB signaling in PS deficiency (Figures 3A–3F and 4I). Sesn2 has been described to be regulated by nuclear calcium (Bading, 2013, Zhang et al., 2009) and to act as negative regulator of mTORC1 by indirectly or directly regulating RagA function (Chantranupong et al., 2014, Peng et al., 2014), largely explaining our data showing excessive binding of mTOR to RagA in PS deficiency (Figures 2E and S3F). Overexpression of Sesn2 rescued CLEAR network activity and mTOR dynamics in PSDKO cells (Figures 3H and 3I) and its binding to HA-RagA (Figure S3F) in PS-deficient human neurons. We conclude that the mTORC1 phenotype we observed in PS deficiency can be largely ascribed to low expression levels of the calcium-regulated gene Sesn2 (see proposed model in Figure 6). The regulation of TFEB through calcineurin (Medina et al., 2015) may present a parallel way to regulate clearance in AD cells. Elevation of endogenous Sesn2 levels resulted in increased autophagy in PS-deficient cells. The effect of CaI on autophagic flux in PS-deficient cells was most obvious in assays in which CQ was used to inhibit lysosomal degradation of LC3-II (Figure 4I, lane 4 versus lane 8).

To understand the physiological consequence of an attenuation of the TFEB-governed CLEAR network, we analyzed the tissues of the brain-specific TFEB KO mice (Tcfeb fl/fl:Nestin-Cre). Neuronal deletion of TFEB resulted in an accumulation of neurotoxic proteins like pTau and amyloid (Figures 5C and 5D). We propose that the buildup of Aβ42 peptides observed in our biochemical studies (Figure S5A) is a consequence of an increase of total Aβ levels leading to possible changes in secretase activities. Similar mechanisms have been proposed for trisomy 21-associated amyloidosis in which one additional copy of the WT APP suffices to induce AD-like phenotypes (Masters et al., 1985, Mégarbané et al., 2009). The observed axonal degeneration and apoptotic cell death correlates with the buildup of neurotoxic proteins. Interestingly, amounts of PHF pTau found in TFEB KO animals showed a stronger association with neuronal death than the increase of the endogenous, murine (not aggregation-prone) Aβ, implying a more direct role of pTau in neurodegeneration.

One important physiological consequence for the attenuation of the CLEAR gene network is the inhibition of autophagy, which is tightly linked to cellular survival under stress conditions. Autophagy inhibition has been directly associated to mTORC1 activity in TSC2-deficient cells (Menon et al., 2014).

Altogether, our data strongly suggest that PS deficiency connects to lysosomal inhibition through a mechanism involving the functional alteration of mTORC1 and the autophagosomal system maintained by TFEB. We present here that the amino acid sensing of mTORC1 is dysregulated in PS deficiency due to low levels of the calcium-regulated gene Sesn2 (Figure 6). Attenuation of the clearance pathways leads to the onset of AD-like phenotypes in mouse brains while PS-deficient cells fail to induce autophagy, eventually exhausting their energy pools and degenerating—a pathway that may likely be one possible explanation for the onset of FAD.

## Experimental Procedures

### Materials

All procedures involving mice were approved by the Institutional Animal Care and Use Committee of the Baylor College of Medicine. All antibodies and reagents used in the presented experiments are described in the Supplemental Experimental Procedures.

### Immunostaining

Cells grown on coverslips were rinsed in PBS once and fixed in 4% paraformaldehyde. Cultures were then rinsed in PBS and blocked in 5% normal goat serum, 0.5% BSA, and 0.5% TritonX100 in PBS for 45 min. Primary antibody working solutions were made in blocking buffer and incubated overnight at 4°C. Cultures were rinsed in PBS and placed in secondary antibodies for 45 min. The specimens were then rinsed and mounted. Confocal images were obtained using a ZEISS spinning-disc microscope.

### Cell Culture

WT and PS1/2 KO MEFs were provided by Bart de Strooper (VIB Center for the Biology of Disease, KU Leuven). MEFs were cultured in DMEM with 10% FBS. Human control and PS1M146L iPSCs were obtained from Scott Noggle (The New York Stem Cell Foundation Research Institute) and previously were characterized extensively (Sproul et al., 2014). Human neuronal cultures were generated in the lab using the previously described dual-Smad inhibition protocol (Chambers et al., 2009).

For the starvation assays, cells were plated at low confluency (20,000 cells/24 wells) on coverslips in 10% FBS-containing DMEM. The next day, fresh media were given and the cells were allowed to stabilize for 2 hr, rinsed twice in HBSS (Gibco) containing calcium and magnesium, and allowed to remain for the indicated time points. For re-feeding experiments, cells were placed in HBSS for the indicated time points and later replaced in nutrient-rich media (10% FBS/DMEM). For leucine/glutamine deprivation experiments, cells were first plated and allowed to stabilize in RPMI 1640 (without leucine and glutamine) containing 10% dFBS along with both leucine and glutamine. For leucine deprivation, the media were then replaced with 10% dFBS in RPMI 1640 without leucine alone for the indicated time points.

### Statistics

The results are given as the mean ± SEM. Statistical analyses were performed with Microsoft Excel using the two-tailed Student’s t test as appropriate. Significant differences of means are indicated as ^∗^p ≤ 0.05, ^∗∗^p ≤ 0.01, and ^∗∗∗^p ≤ 0.005.

## Author Contributions

K.R. conducted experiments, evaluated data, and wrote parts of the manuscript. C.L.C., K.K., I.C.N., C.S., and T.B.H. conducted experiments and collected data shown in this study. S.A.N. provided AD iPSC lines and corrected the manuscript. A.B. provided the CLEAR-luciferase constructs and the TFEB KO tissues and corrected the manuscript. R.D. advised the team, designed and conducted experiments, evaluated data, and wrote the manuscript.

## Supplemental Information

Document S1. Supplemental Experimental Procedures and Figures S1–S5

Document S2. Article plus Supplemental Information
