# Supplementary material for: Dysregulation of Nutrient Sensing and CLEARance in Presenilin Deficiency
Source: Cell Rep. 2016 Feb 25;14(9):2166–79. doi: 10.1016/j.celrep.2016.02.006 (PMC4793148; doi:10.1016/j.celrep.2016.02.006)
Supplement: Document S1. Supplemental Experimental Procedures and Figures S1–S5 [file mmc1.pdf]

Cell Reports, Volume *14*

Supplemental Information

Dysregulation of Nutrient Sensing and CLEARance in Presenilin Deficiency

Kavya Reddy, Corey L. Cusack, Israel C. Nnah, Khoosheh Khayati, Chaitali Saqcena, Tuong B. Huynh, Scott A. Noggle, Andrea Ballabio, and Radek Dobrowolski

Figure S1

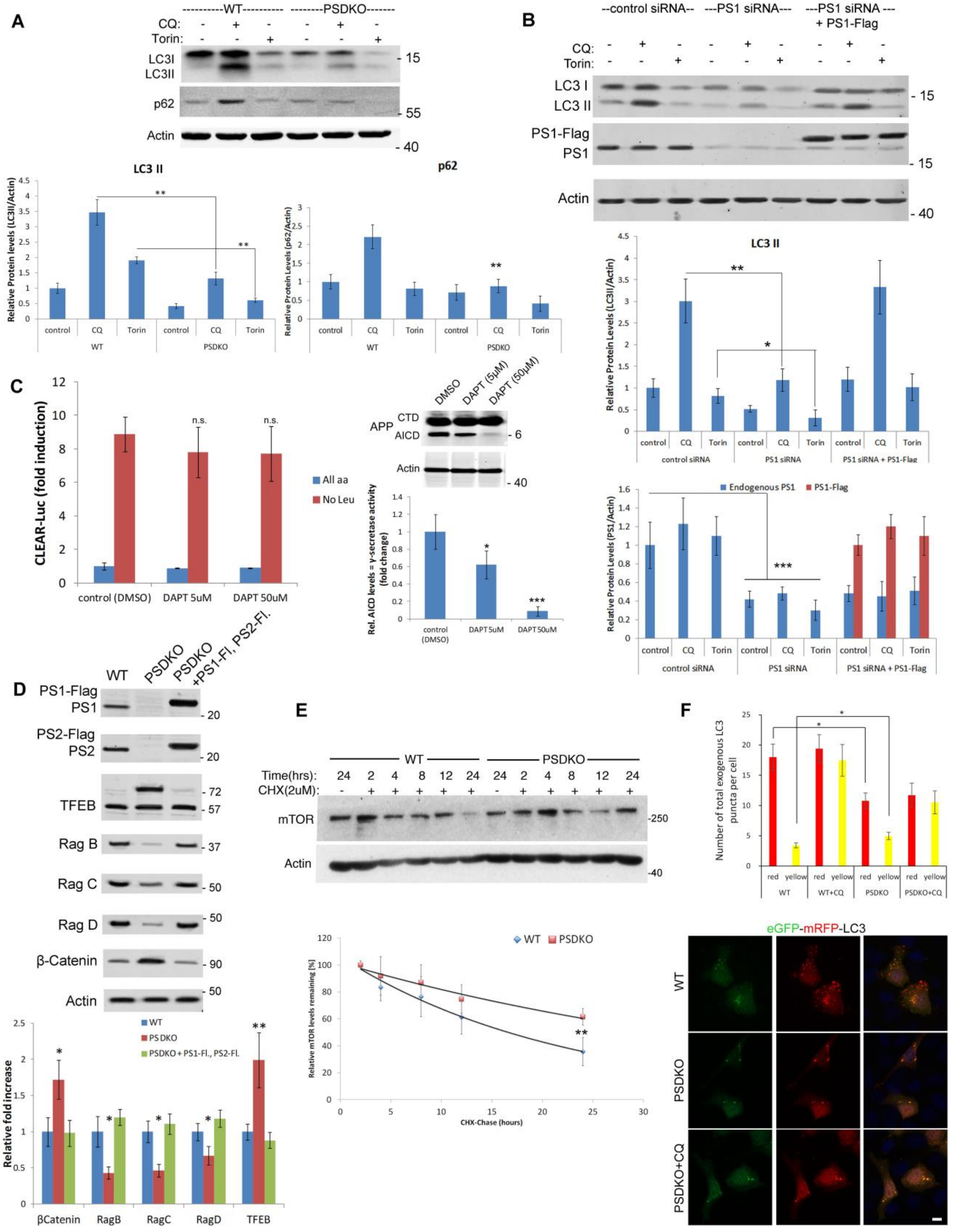
A
----------WT---------- --------PSDKO-------
CQ:
Torin:
LC3I
LC3II
p62
Actin
LC3 II
p62
Relative Protein levels (LC3II/Actin)
Relative Protein Levels (p62/Actin)
control CQ Torin control CQ Torin
WT
PSDKO
B
--control siRNA-- ---PS1 siRNA--- ---PS1 siRNA --- + PS1-Flag
LC3 I
LC3 II
PS1-Flag
PS1
Actin
LC3 II
Relative Protein Levels (LC3II/Actin)
control siRNA
PS1 siRNA
PS1 siRNA + PS1-Flag
Endogenous PS1
PS1-Flag
Relative Protein Levels (PS1/Actin)
C
CLEAR-Luc (fold induction)
n.s.
control (DMSO)
DAPT 5uM
DAPT 50uM
All aa
No Leu
DMSO
DAPT (5uM)
DAPT (50uM)
APP
CTD
AICD
Actin
Rel. AICD levels = γ-secretase activity (fold change)
D
WT
PSDKO
PSDKO +PS1-Fl, PS2-Fl.
PS1-Flag
PS1
PS2-Flag
PS2
TFEB
Rag B
Rag C
Rag D
β-Catenin
Actin
PSDKO + PS1-Fl., PS2-Fl.
Relative fold increase
βCatenin
RagB
RagC
RagD
TFEB
E
Time(hrs):
CHX(2uM):
mTOR
Actin
Relative mTOR levels remaining [%]
CHX-Chase (hours)
F
Number of total exogenous LC3 puncta per cell
red
yellow
WT+CQ
PSDKO+CQ
eGFP-mRFP-LC3

Figure S1. Mechanistic Target of Rapamycin Complex 1 (mTORC1) Is Deregulated in Presenilin Deficiency, Attenuating the CLEAR Network Activity. Related to Figure 1.

(A) Expression of low levels of LC3 and p62 in PSDKO cells in an autophagy flux assay. WT and PSDKO cells were treated with CQ (25 µM) or Torin (250 nM) for 8hrs to inhibit lysosomal function or induce autophagy through mTOR inhibition, respectively. Low levels of LC3 (3.47±0.4 vs 1.32±0.2, p=0.006) and p62 (2.21±0.3 vs 0.88±0.2, p=0.005) even after CQ treatment indicate an attenuation of autophagy function. CQ treatment is not affecting p62 levels in PSDKO cells, while being sensitive to Torin1 treatment.

(B) Isogenic, iPSC-derived human neurons depleted from PS1 show low autophagy flux (LC3II levels) compared to controls (3.01±0.5 vs 1.18±0.3, p=0.005). Autophagy flux is normalized to control levels when PS1-Flag is expressed in PS1 knock-down neurons (3.01±0.5 vs 3.33±0.6, p=0.1). Immunoblot analyses confirmed successful depletion of endogenous PS1 (1.0±0.2 vs 0.4±0.08, p= 0.005) and expression of PS1-Flag in these cultures.

(C) Pharmacological inhibition of γ-secretase activity with Difluorophenacetyl-L-alanyl-S-phenylglycine t-butyl ester (DAPT) does not significantly change CLEAR-luciferase activity in 293T cells. Gamma-secretase activity is indirectly represented by the amount of APP intracellular domain (AICD).

(D) Re-expression of PS1-Flag and PS2-Flag in PSDKO MEFs normalizes the levels of β-Catenin, Rag B, Rag C, Rag D, and TFEB (β-Catenin: PSDKO 1.72±0.27 vs PSDKO+PS1,2Flag 0.98±0.18; Rag B: PSDKO 0.43±0.08 vs PSDKO+PS1,2Flag 1.2±0.1; Rag C: PSDKO 0.46±0.1 vs PSDKO+PS1,2Flag 1.1±0.14; Rag D: PSDKO 0.67±0.12 vs PSDKO+PS1,2Flag 1.2±0.1; and TFEB: PSDKO 1.99±0.37 vs PSDKO+PS1,2Flag 0.88±0.11).

(E) mTOR stability is increasd in cycloheximide (CHX) pulse-chase assays. Representative image showing a immunoblot analysis and quantification of n=3 analyses show a delayed mTOR degradation in PSDKO cells, reaching significance after 24 hours of chase (24 h time point: WT: 35.7%±6.1 vs PSDKO: 61.5±10.5, p=0.01). The half-life time of the mTOR protein was calculated to $t_{1/2,\ WT}$=16h for WT cells, and to $t_{1/2,\ PSDKO}$=30h in PSDKO cells.

(F) Baseline autophagy flux is impaired in PSDKO cells as detected using the exogenous eGFP-mRFP-LC3 reporter constructs. Here, the number of yellow puncta (= not acidified LC3-positive organelles) is increased in PSDKO in comparison to WT cells (WT: 3±0.4 vs PSDKO: 5±0.6, p=0.04). The number of red puncta is decreased in PSDKO cell (WT: 18±2.1 vs PSDKO: 11±1.3, p=0.04). Lysosomal inhibition with Chloroquine significantly increases the number of yellow LC3 puncta in both cell lines (WT: 17%±2.6 vs PSDKO: 12±1.9, p=0.1).

Scale bar=10µm.

Data are represented as mean ± SEM.

Figure S2

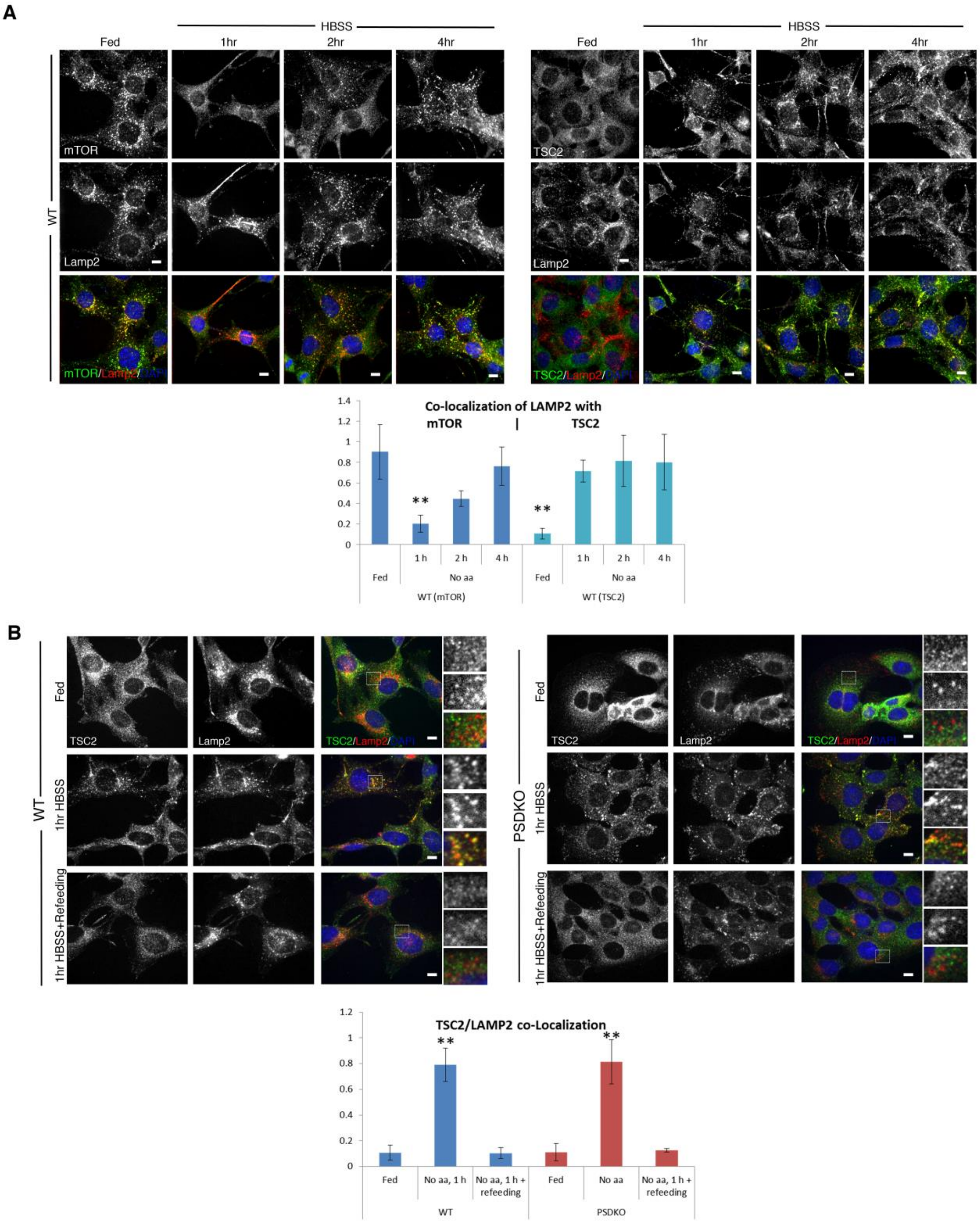
A
HBSS
Fed
1hr
2hr
4hr
WT
mTOR
Lamp2
mTOR/Lamp2/DAPI
TSC2
TSC2/Lamp2/DAPI
Co-localization of LAMP2 with
mTOR | TSC2
1.4
1.2
1
0.8
0.6
0.4
0.2
0
**
1 h
2 h
4 h
Fed
No aa
WT (mTOR)
WT (TSC2)
B
Fed
1hr HBSS
1hr HBSS+Refeeding
PSDKO
TSC2/LAMP2 co-Localization
No aa, 1 h
No aa, 1 h + refeeding
No aa
WT
PSDKO

Figure S2. Defective amino acid sensing of mTORC1 in PSDKO Cells is Mediated by Excessive RagA binding And Independent of TSC2 Localization. Related to Figure 2.

(A) mTOR re-localizes back to LAMP2 vesicles at 4hrs of starvation, and is found on lysosomes at the same time as TSC2. WT cells were starved for 1hr, 2hr and 4hr in HBSS and the localization of mTOR/TSC2 and Lamp2 was determined by immunostaining. Quantification of lysosomal co-localization: at 1 h time point: mTOR 0.2±0.08 vs TSC2 0.7±0.1, at 4 h time point: mTOR: 0.76±0.2 vs TSC2: 0.8±0.3.

(B) TSC2 localization upon starvation and re-feeding is normal in PSDKO cells. WT and PSDKO cells were starved for 1hr in HBSS and re-fed with nutrient rich media. Insets depict selected fields that were magnified. Quantification of lysosomal co-localization, 1 h after aa starvation WT: 0.79±0.1 vs PSDKO: 0.8±0.2, 1h after aa re-feeding WT 0.1±0.04 vs PSDKO 0.12±0.01.

Scale bar=10μm.

Data are represented as mean ± SEM.

Figure S3

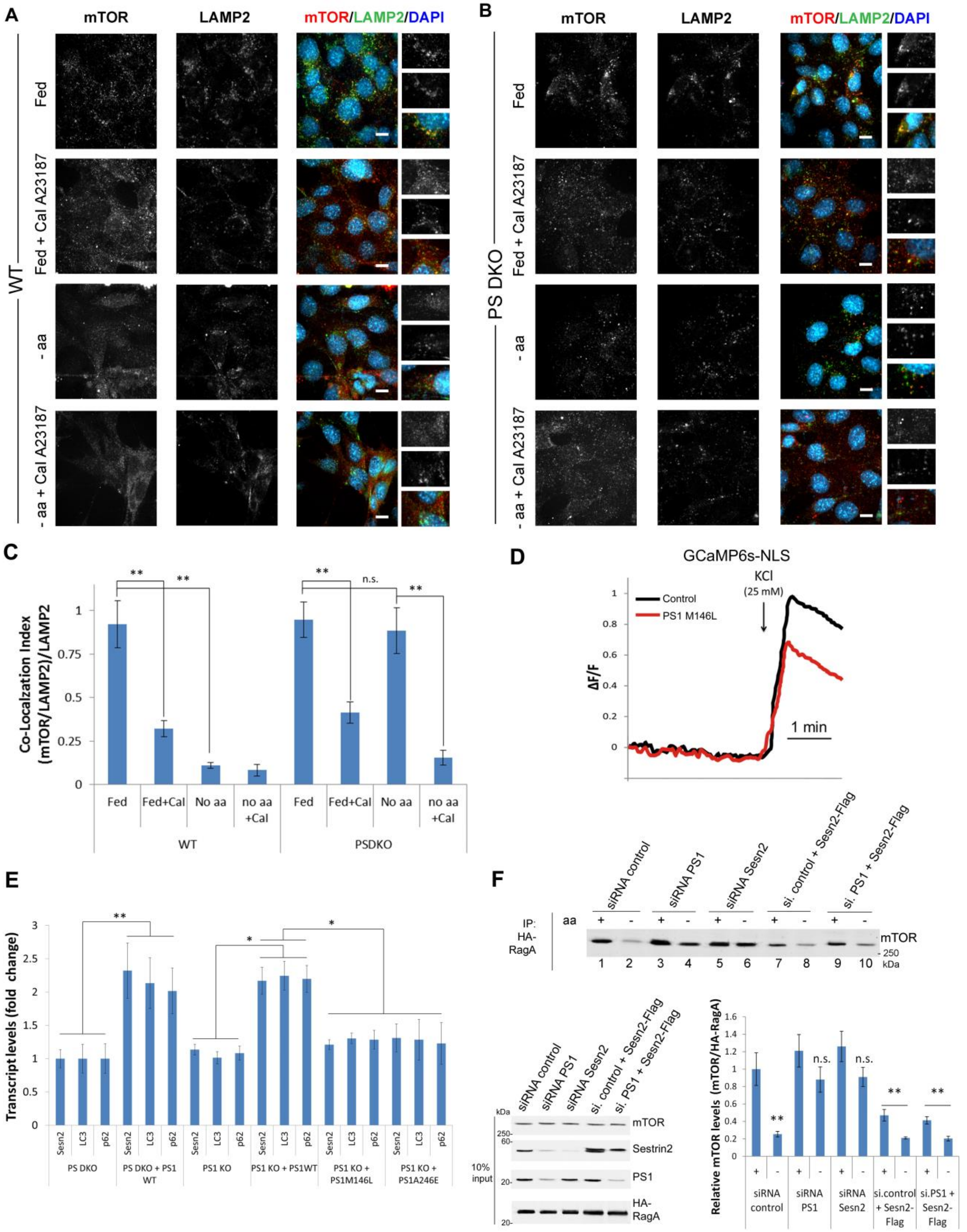
A
B
mTOR
LAMP2
mTOR/LAMP2/DAPI
WT
PS DKO
Fed
Fed + Cal A23187
- aa
- aa + Cal A23187
C
Co-Localzation Index (mTOR/LAMP2)/LAMP2
Fed
Fed+Cal
No aa
no aa +Cal
WT
PSDKO
n.s.
D
GCaMP6s-NLS
Control
PS1 M146L
KCl (25 mM)
ΔF/F
1 min
E
Transcript levels (fold change)
Sesn2
LC3
p62
PS DKO
PS DKO + PS1 WT
PS1 KO
PS1 KO + PS1WT
PS1 KO + PS1M146L
PS1 KO + PS1A246E
F
siRNA control
siRNA PS1
siRNA Sesn2
si. control + Sesn2-Flag
si. PS1 + Sesn2-Flag
IP: HA-RagA
aa
mTOR
250 kDa
10% input
Sestrin2
PS1
HA-RagA
Relative mTOR levels (mTOR/HA-RagA)
si.control + Sesn2-Flag
si.PS1 + Sesn2-Flag

Figure S3. Low Nuclear Calcium Levels Are Responsible For Low Sestrin 2 Levels and mTOR Dysregulation In PS Deficient Cells. Related to Figure 3.

(A and B) Increase of cellular calcium rescues mTOR amino acid sensing in PSDKO cells. PSDKO cells were pretreated with Calcium Ionophore (CI; A23187) at 5 μM for 4hrs, followed by HBSS treatment for 1hr. The localization of mTOR and LAMP2 was determined by immunostaining in WT cells. Insets depict magnified areas from individual channels.

(C) Quantification of calcium ionophore experiments presented in Figures S3A and S3B. Level of co-localization of mTOR has been determined by assessing the amount of mTOR/LAMP2 fluorescence intensities divided by LAMP2 signal intensity (mTOR/LAMP2)/LAMP2. While mTOR and LAMP2 signals highly co-localize in fed conditions in WT cells (0.92±0.13) and fed and starvation conditions in PSDKO cells (0.94±0.1 and 0.88±0.13, p=0.1, n.s.), an increase of cellular calcium disperses mTOR from the LAMP2-positive compartment in both cell lines (WT: 0.32±0.05, PSDKO:0.4±0.06, p=0.01 on bracket), while amino acid starvation was only effective in WT cells (0.11±0.02, p=0.008 on bracket).

(D) Representative tracings of the human synapsin promoter driven GCaMP6s-NLS calcium biosensor activity in human neurons (control=black line, PS1M146L=red line). iPSC-derived human neurons were transfected with GCaMP6s-NLS for live imaging next day. Cells were imaged in Neuro-culture medium and at indicated time point (arrow), KCl (25 mM) was added to induce a calcium peak. Note the relatively low amplitude of the GCaMP6s-NLS signal in PS1M146L cells when compared to controls. Plots representing an average cytosolic and nuclear calcium peak values are shown in the main Figure 3B.

(E) Decreased transcription of Sesn2, LC3 and p62 in PS 1 and PS1,2 double knock-out cells can be elevated by expressing exogenous PS1WT-Flag constructs while AD-associated PS1 mutations did not have such an effect. Sesn2 mRNA levels are as follows: PSDKO: 1±0.1 vs PSDKO+PS1WT-Flag: 2.3±0.4, p=0.01; PS1KO 1.1±0.1 vs PS1KO+PS1WT-Flag: 2.2±0.2, p=0.05; PS1KO+PS1WT-Flag vs PS1M146L 1.2±0.1, PS1A246E 1.3±0.1, p=0.05.

(F) Co-immunoprecipitation analyses assessing binding of endogenous mTOR proteins to HA-RagA under starvation conditions in iPSC-derived human neurons. While mTOR dissociates from HA-RagA under amino acid starvation in control cells (fed: 1±0.2vs starved: 0.25±0.03, p=0.01), mTOR remain strongly bound to HA-RagA under starvation in PS1 (0.88±0.1, p=0.1 when compared to starvation in control samples) or Sesn2 (0.9±0.1, p=0.1 to control) depleted human neurons. Overexpression of Sesn2-Flag reduces the baseline binding of mTOR to HA-RagA in control siRNA (0.47±0.1, p=0.01 to fed control) or PS1 depleted cells (0.4±0.04, p=0.01 to fed control), and rescues the excessive mTOR-RagA binding in PS1 deficient cells under starvation (0.2±0.02, p=0.05 to starved PS1 siRNA samples).

Scale bar=10μm.

Data are represented as mean ± SEM.

Figure S4

A

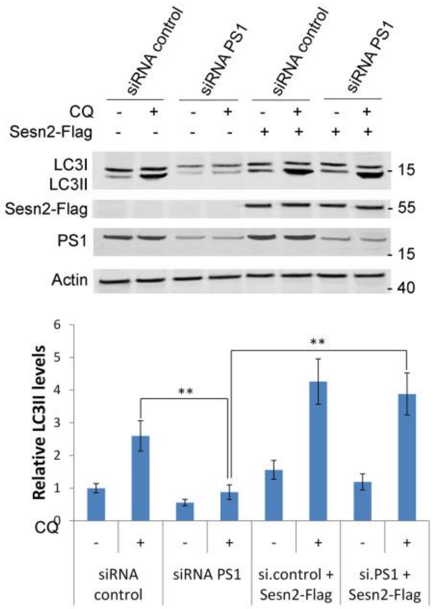

siRNA control
siRNA PS1
siRNA control
siRNA PS1
CQ
Sesn2-Flag
LC3I
LC3II
- 15
Sesn2-Flag
- 55
PS1
- 15
Actin
- 40
Relative LC3II levels
**
**
CQ
siRNA control
siRNA PS1
si.control + Sesn2-Flag
si.PS1 + Sesn2-Flag

Figure S4. TFEB Driven Clearance Functions Are Impaired Due To Low Sestrin 2 Levels In Presenilin Deficient Cells. Related to Figure 4.

(A) Expression of exogenous Sestrin2-Flag increases autophagy flux in control siRNA and normalizes autophagy levels in PS1 depleted human neurons. iPSC-derived human neurons were depleted from PS1 (72%±12% knock-down efficiency) and reversely transfected with Sesn2-Flag constructs which expression was evaluated in anti-Flag (Sesn2-Flag) immunoblot analyses. In autophagy flux assays, in conditions when lysosomes are inhibited (CQ treatment), LC3II levels increase when Sesn2-Flag is expressed, largely eliminating the differences between control and PS deficient neurons (siRNA control+Sesn2-Fl: 4.2±0.7 vs siRNA PS1+Sens2-Fl: 3.9±0.6, p=0.1), indicating restored autophagy flux.

Data are represented as mean ± SEM.

Figure S5

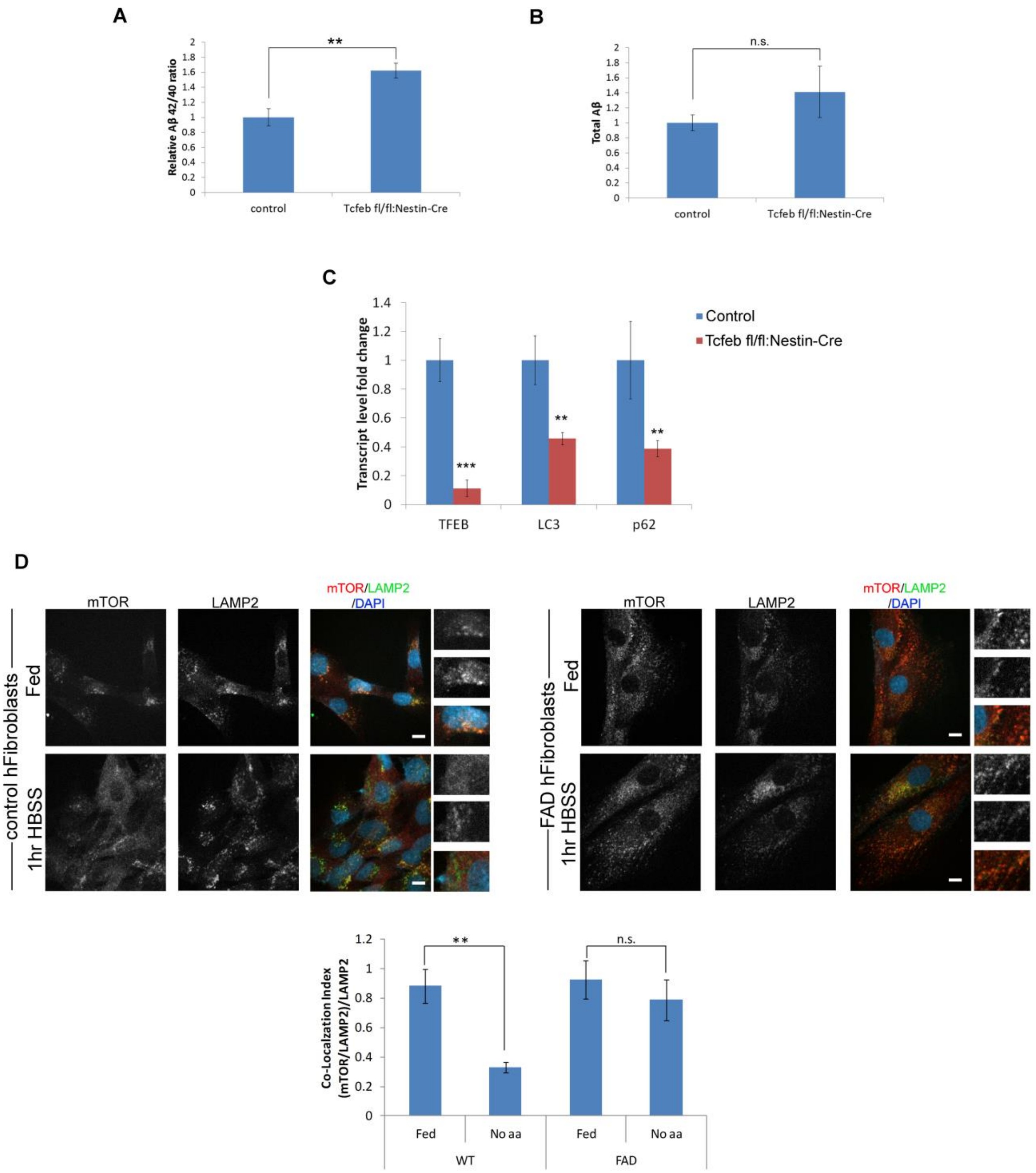
A
**
Relative Aβ 42/40 ratio
control
Tcfeb fl/fl:Nestin-Cre
B
n.s.
Total Aβ
control
Tcfeb fl/fl:Nestin-Cre
C
Control
Tcfeb fl/fl:Nestin-Cre
Transcript level fold change
***
**
**
TFEB
LC3
p62
D
mTOR
LAMP2
mTOR/LAMP2/DAPI
control hFibroblasts
Fed
1hr HBSS
FAD hFibroblasts
Co-Localzation Index (mTOR/LAMP2)/LAMP2
n.s.
Fed
No aa
WT
FAD

Figure S5. Cellular Clearance Is Attenuated in AD Cells and Leads to AD-Like Phenotypes In Vivo. Related to Figure 5.

(A) Relative Aβ 42/40 ratios were determined in Tcfeb fl/fl:Nestin-Cre whole brain lysates. The conditional TFEB knock-out tissues displayed higher Aβ 42/40 ratios than control littermates (1.6 fold ± 0.98, $p=0.007$).

(B) Analyses of total Aβ levels in Tcfeb fl/fl:Nestin-Cre whole brain lysates indicated a trend of increased total Aβ levels (1.4 fold ± 0.34, $p=0.06$).

(C) Quantification of total transcript levels of TFEB, p62, and LC3 in Tcfeb fl/fl:Nestin-Cre whole brain lysates.

(D) Control hFibroblasts disperse the lysosomal mTORC1 under amino acid starvation (1h HBSS treatment), while primary hFibroblasts from a FAD patient show extensive tethering of the complex on expanded lysosomal compartment (LAMP2). Quantitative evaluation of lysosomal mTOR in amino acid starvation assays revealed a low mTOR-LAMP2 co-localization and an excessive mTOR tethering to lysosomes in FAD fibroblasts (no aa in WT: 0.33±0.04 and no aa in FAD: 0.8±0.1, $p=0.1$).

Scale bar=10μm.

Data are represented as mean ± SEM.

SUPPLEMENTAL EXPERIMENTAL PROCEDURES

Materials

The following primary antibodies were used for immunoblotting and immunostaining: mTOR (#2983; Cell Signaling), TSC2 (#4308, Cell Signaling), Rheb (E1G1R, Sample aliquot for testing from Cell Signaling), P-p70S6K (Thr389, #9205, Cell Signaling), P70S6K (#2708, Cell Signaling), RagA, RagB, RagC, RagD (#9778, Cell Signaling, Rag antibody sampler kit), mouse LAMP2 (#ABL-93,DSHB), human LAMP2 (#H4B4), LC3 a/b (#4108, Cell Signaling) p62/SQSTM1 (GP62-C, Progen), TFEB (#ab2636, Abcam; for immunoblotting),TFEB (#MBS120432 for immunostaining), β-Actin (#JLA20, DSHB), β-Catenin (#CC206, Sigma), CREB (#9197, Cell Signaling), pCREB (#9191, Cell Signaling), Sestrin2 (#10795-1-AP, ProteinTech), Presenilin 1 (#3622, Cell Signaling), Presenilin2 (#2192, Cell Signaling), MAP2 (#ab5392, Abcam), βIII-Tubulin (#MMS-435P, Covance), cleaved Caspase3 (#9661, Cell Signaling). Secondary antibodies coupled to either Infrared Dyes (IRDye 680 and IRDye 800, LICOR), or to HRP (Jackson IR) were used for immunoblotting analysis, those coupled to fluorochromes (Jackson IR) were used for immunostaining. Calcium Ionophore (A23187, #C7522) and DAPT (#D5942) were purchased from Sigma. RPMI media without Leucine/L-Glutamine (#R899912, USBiological), HBSS 10X (#14065-056, Life Technologies), and L-Leucine(#L800) were from Sigma. For knock-down experiments, the following siRNA reagents were used: ON-TARGET plus SMARTpool siRNA for Sesn2 (E-052642-00), CaMKIV (E-004944-00), CREB (E-003619-00), PS1 (E-004998-00), PS2 (E-006018-00), and Control (D-001810) siRNA were purchased from GE Dharmacon. siRNAs were transfected as described using the Lipofectamine RNAimax reagent protocol, an efficient knock-down of gene expression was validated in qPCR or immunoblot analyses.

SDS PAGE, Western Blot, and Pulse-Chase Analyses

Cell lysates were prepared with lysis buffer (10% glycerol, 1% NP40, 20mM Tris (pH7.4), 2.5 mM EDTA (pH8), 2.5mM EGTA (pH8), including Roche Protease inhibitor cocktail (#04693116001). Lysates were spun down at 14000rpm for 15´ at 4°C. The cleared supernatant was collected and protein concentration assayed using the BCA method and run on a 10-12% SDS-PAGE gels. The protein was then transferred onto PVDF (Immobilon) membranes. The membrane was blocked in 5% Milk, 0.1% TritonX 100 in TBS for 45´. After incubation with primary antibodies (overnight) and secondary antibodies (45´), the membranes were washed and analyzed by either the LI-COR Odyssey system or developed by ECL (Pierce).

To determine protein stability of mTOR in WT and PSDKO cells, both cell lines were cultured to a confluency of up to 90% and incubated with Cycloheximide (CHX, Sigma-Aldrich) for the indicated time-points to inhibit further protein translation. Protein lysates were made, pre-cleared and used for western blot analyses as described above. Band intensities indicating for example total mTOR and βActin amounts were quantified, ratios of these intensities (mTOR/Actin) for at least three independent experiments.

Co-Immunoprecipitation

Mouse embryonic fibroblasts or human neuronal precursor cells were reversely transfected either with HA-RagA or HA-Rheb (Sancak et al., 2008), let reach confluency overnight, and use for the experiment or let differentiate into human neurons for approx. 7 days. Cells were lysed in IP-buffer (50 mM Tris, 150 mM NaCl, 10 mM EDTA, 1% NaF, 1% Na3VO4, 1% NP40, 0.5 mM DTT, 1x Complete Protease Inhibitor Cocktail (Roche)). Lysates were pre-cleared by centrifugation and incubated with anti-HA antibodies in the cold, overnight. MagnaBind goat anti-rabbit beads (ThermoScientific) were used to co-immunoprecipate proteins binding to HA-RagA or HA-Rheb at a rotating mixer overnight. Loaded beads were washed with the IP-buffer at least 3 times, resuspended in Laemmli buffer, and boiled for 10 min. Eluted proteins and protein input were used in western blot analyses to determine binding efficiencies of endogenous mTOR and TSC2 proteins to HA-RagA and HA-Rheb in both cell lines under starvation conditions.

Amino acid starvation experiments

For the starvation assays, cells were plated at low confluency (20,000 cells / 24 well) on coverslips in 10% FBS containing DMEM. The next day, fresh media was given and the cells allowed to stabilize for 2hrs, rinsed twice in Hank's Balanced Salt Solution (HBSS, Gibco) containing calcium and magnesium, and allowed to remain for the indicated time points. For re-feeding experiments, cells were placed in HBSS for the indicated time points and later

replaced in nutrient rich media – 10%FBS/DMEM. For leucine/glutamine deprivation experiments, cells were first plated and allowed to stabilize in RPMI 1640 (without Leucine and Glutamine) containing 10% dFBS along with both Leucine and Glutamine. For Leucine deprivation, the media was then replaced with 10% dFBS in RPMI1640 without leucine alone for the indicated time points. For re-stimulation experiments, cells were grown as above and starved for 1hr with HBSS and then re-stimulated with media containing Leucine or nutrient rich media.

Plasmid DNA constructs and transfections

4XCLEAR Luciferase construct was obtained from Andrea Ballabio (Sardiello et al., 2009). SV40 Renilla Luciferase (Chen and Prywes, 1999) was used to normalize firefly luciferase values for transfection efficiency (resulting values are expressed as relative luciferase units, RLUs). PS1/2-Flag constructs are described in Dobrowolski et al., 2012. TFEB-(carboxy)-1xFlag was cloned by amplifying the human TFEB sequence out of the corresponding cDNA sequence (OriGene), Sestrin1, 2, 3, RagA-HA, Rheb-HA were obtained from David Sabatini (Chantranupong et al., 2014; Sancak et al., 2010). ptfLC3 was a gift from Tamotsu Yoshimori (Addgene plasmid # 21074), pcDNA3 Flag TSC2 was a gift from Brendan Manning (Addgene plasmid # 14129, Manning et al., 2002). All constructs were transfected using BioT plasmid transfection protocol (Bioland Scientific).

GCaMP6s reporter assays

GCaMP6s (Chen et al., 2013) and GCaMP6s-NLS (Hagenston and Bading, 2011 and unpublished data) reporter were used to determine the level of cytosolic and nuclear calcium signaling, respectively. Both genetically encoded reporters were transfected separately into iPSC-derived human neurons (control or including the PS1M146L mutation). Baseline fluorescence and KCl (25 mM) triggered GCaMP-fluorescence was measured in spinning disc confocal microscopy over the time. Differences in fluorescence were divided by baseline fluorescence intensities and plotted as function over time. Minima and maxima intensities were normalized to 0 or 1, respectively.

Luciferase Assays

4xCLEAR Luciferase assays have been used to representatively assess the activity of the TFEB-regulated genes, the so called CLEAR gene network (Sardiello et al., 2009). In this assay, binding of TFEB to the 4x repeated CLEAR-response element promotes the expression of luciferase which levels are determined in Dual-Luciferase Reporter assays. Cells were plated in 24 well tissue culture plates in equal numbers. 4XCLEAR Luciferase and SV40 Renilla luciferase plasmids were transfected using BioT transfection reagent. SV40 Renilla luciferase was used to normalize for transfection and expression efficiencies. Wherever indicated other constructs were also co-transfected following manufacturer's manual. Twenty four hours after transfection the cells were harvested in passive lysis buffer and 10 ul of the pre-cleared lysate used to assay luciferase activity using the Promega Dual-Luciferase Reporter kit. In other experiments, Sesn2-Luciferase constructs were used to detect the promotor activity of the human Sestrin2 gene.

Quantitative PCR analyses

For RNA isolation WT and PSDKO cells were plated at 75% confluency and let grow over night. Total RNA was prepared using the TRIZOL reagent (LifeTech) according to manufacturer's instructions followed by a chloroform wash and ethanol precipitation. Two µg of total RNA was used for cDNA synthesis using the Single Strand cDNA Synthesis kit (Fermentas). Copy-DNA was diluted 10-fold and used for qPCR analyses using SYBRgreen (ABiosystems) and the Roche Lightcycler 480. Primer sequences from the PGA-PrimerBank were used. Following primer sequences were used for the presented analyses: LC3_for: CGCTTGCAGCTCAATGCTAAC, LC3_rev: CTCGTACACTTCGGAGATGGG; p62_for GAACTCGCTATAAGTGCAGTGT, p62_rev: AGAGAAGCTATCAGAGAGGTGG; Sesn2_for: TCCGAGTGCCATTCCGAGAT, Sesn2_rev: TCCGGGTGTAGACCCATCAC; TFEB_for: GCAGCCACCTGAACGTGTA; TFEB_rev: TGTTAGCTCTCGCTTCTGAGT; Actin_for: TTGCTGACAGGATGCACGAAG, Actin_rev: CCACCGATCCACACAGAGTA.

EGFP-mRFP-LC3 Assays

For assays showing the levels of the EGFP-mRFP-LC3 biosensor, ptfLC3 construct has been transiently transfected into WT and PSDKO mouse embryonic fibroblasts cultured on fibronectin coated cover glass. Next day, the medium was changed and the cells were left to recover after transfection for 24-36 hours. Some of the cells were incubated

with 100 mM Chloroquine for 1 hour to inhibit lysosomes and thereby autophagy flux. Cells were fixed with 4% paraformaldehyde, washed in PBS+ before and after fixation, mounted and imaged the same day. Confocal imaging was performed using the Zeiss spinning disk confocal microscope. At least 100 cells expressing red puncta (mRFP-LC3 signal) were imaged, the GFP signal was recorded blindly. Red and yellow puncta were counted per cell, the yellow signal indicated the existence of not acidified, immature autophagosomes. The experiments were repeated 3 times.

Aβ Assays

Brains of control and Tcfeb fl/fl:Nestin-Cre mice were shock-frozen in liquid nitrogen, pulverized and reconstituted in standard lysis buffer. Human/rat (mouse) Aβ 1-40 and 1-42 ELISA kits (Wako) were used according to the manufacturer's instructions to quantify Aβ levels. Brain samples of two mice from each genotype were used for these assays. All assays were performed blindly, repeated 3 times and averaged for each triplicate.

SUPPLEMENTAL REFERENCES

Chantranupong, L., Wolfson, R.L., Orozco, J.M., Saxton, R.A., Scaria, S.M., Bar-Peled, L., Spooner, E., Isasa, M., Gygi, S.P., and Sabatini, D.M. (2014). The Sestrins Interact with GATOR2 to Negatively Regulate the Amino-Acid-Sensing Pathway Upstream of mTORC1. Cell Rep. *9*, 1–8.

Chen, X., and Prywes, R. (1999). Serum-induced expression of the cdc25A gene by relief of E2F-mediated repression. Mol. Cell. Biol. *19*, 4695–4702.

Chen, T.-W., Wardill, T.J., Sun, Y., Pulver, S.R., Renninger, S.L., Baohan, A., Schreiter, E.R., Kerr, R.A., Orger, M.B., Jayaraman, V., et al. (2013). Ultrasensitive fluorescent proteins for imaging neuronal activity. Nature *499*, 295–300.

Hagenston, A.M., and Bading, H. (2011). Calcium signaling in synapse-to-nucleus communication. Cold Spring Harb. Perspect. Biol. *3*, a004564.

Manning, B.D., Tee, A.R., Logsdon, M.N., Blenis, J., and Cantley, L.C. (2002). Identification of the tuberous sclerosis complex-2 tumor suppressor gene product tuberin as a target of the phosphoinositide 3-kinase/Akt pathway. Mol. Cell *10*, 151–162.

Sancak, Y., Peterson, T.R., Shaul, Y.D., Lindquist, R.A., Thoreen, C.C., Bar-Peled, L., and Sabatini, D.M. (2008). The Rag GTPases bind raptor and mediate amino acid signaling to mTORC1. Science *320*, 1496–1501.

Sancak, Y., Bar-Peled, L., Zoncu, R., Markhard, A.L., Nada, S., and Sabatini, D.M. (2010). Ragulator-rag complex targets mTORC1 to the lysosomal surface and is necessary for its activation by amino acids. Cell *141*, 290–303.

Sardiello, M., Palmieri, M., di Ronza, A., Medina, D.L., Valenza, M., Gennarino, V.A., Di Malta, C., Donaudy, F., Embrione, V., Polishchuk, R.S., et al. (2009b). A gene network regulating lysosomal biogenesis and function. Science *325*, 473–477.
